# Crystal structures of two Co(NCS)_2_ urotropine coordination compounds with different Co coordinations

**DOI:** 10.1107/S2056989022001037

**Published:** 2022-02-03

**Authors:** Christoph Krebs, Inke Jess, Christian Näther

**Affiliations:** a Institute of Inorganic Chemistry, University of Kiel, Max-Eyth.-Str. 2, 24118 Kiel, Germany

**Keywords:** crystal structure, cobalt thio­cyanate, urotropine, hydrogen bonding, mixed ligand occupation

## Abstract

The reaction of Co(NCS)_2_ with urotropine in ethanol leads to the formation of two different compounds, namely, bis­(ethanol-κ*O*)bis­(hexa­methyl­ene­tetra­mine-κ*N*)bis­(thio­cyanato-κ*N*)cobalt(II)–di­aqua-κ*^2^O*-bis­(hexa­methyl­ene­tetra­mine-κ*N*)bis­(thio­cyanato-κ*N*)cobalt(II)–ethanol–hexa­methyl­ene­tetra­mine (1.2/0.8/1.6/4), [Co(NCS)_2_(C_6_H_12_N_4_)_2_(C_2_H_6_O)_2_]_1.2_·[Co(NCS)_2_(C_6_H_12_N_4_)_2_(H_2_O)_2_]_0.8_·1.6C_2_H_6_O·4C_6_H_12_N_4_, **1**, and tris­(ethanol-κ*O*)(hexa­methyl­ene­tetra­mine-κ*N*)bis(thio­cyanato-κ*N*)cobalt(II), [Co(NCS)_2_(C_6_H_12_N_4_)(C_2_H_6_O)_3_], **2**. In the crystal structure of compound **1**, two crystallographically independent discrete complexes are observed that are located on centres of inversion. In one of them, the Co cation is octa­hedrally coordinated to two terminal N-bonded thio­cyanate anions, two urotropine ligands and two ethanol mol­ecules, whereas in the second complex 80% of the coordinating ethanol is exchanged by water. Formally, compound **1** is a mixture of two different complexes, *i.e.* di­aqua­dithio­cyanato­bis­(urotropine)cobalt(II) and di­ethano­ldi­thio­cyanato­bis­(uro­trop­ine)cobalt(II), that contain additional ethanol and urotropine solvate mol­ecules leading to an overall composition of [Co(NCS)_2_(urotropine)_2_(ethanol)_1.2_(H_2_O)_0.8_·0.8ethanol·4urotropine. Both discrete complexes are linked by inter­molecular O—H⋯O and O—H⋯N hydrogen bonding and additional urotropine solvate mol­ecules into chains, which are further connected into layers. These layers combine into a three-dimensional network by pairs of centrosymmetric inter­molecular C—H⋯S hydrogen bonds. In the crystal structure of compound **2**, di­thio­cyanato­(urotropine)tri­ethano­lcobalt(II), the cobalt cation is octa­hedrally coordinated to two terminal N-bonded thio­cyanate anions, one urotropine ligand and three ethanol mol­ecules into discrete complexes, which are located in general positions. These complexes are linked by inter­molecular O—H⋯N hydrogen bonding into layers, which are further connected into a three-dimensional network by inter­molecular C—H⋯S hydrogen bonding.

## Abstract 2

In the crystal structure of the title compounds, discrete complexes with different Co coordinations are observed, which are linked by inter­molecular hydrogen bonding into three-dimensional networks.

## Chemical context

Recently, we reported the crystal structure of two new coordination compounds with the composition [Co(NCS)_2_(urotropine)_2_(ethanol)_2_] and [Co(NCS)_2_(ethanol)_4_](urotropine)_2_ (Krebs *et al.*, 2022 ▸). Both compounds consist of discrete complexes, in which the cobalt cations are octa­hedrally coordinated by two terminal N-bonded thio­cyanate anions and by four ethanol and two ethanol and two urotropine ligands, respectively. These investigations were performed to prepare precursors that on thermal decomposition transform into coordination polymers in which the cobalt cations are linked by μ-1,3 bridging thio­cyanate anions into chains or layers (Näther *et al.*, 2013 ▸). Several such compounds have been reported in the literature and they are of inter­est because they show ferromagnetic or anti­ferromagnetic ordering or a slow relaxation of the magnetization, which is indicative for single-chain magnetism (Böhme *et al.*, 2020 ▸; Shi *et al.*, 2006 ▸; Jin *et al.*, 2007 ▸; Jochim *et al.*, 2020 ▸; Prananto *et al.*, 2017 ▸; Mautner *et al.*, 2018 ▸; Rams *et al.*, 2020 ▸; Ceglarska *et al.*, 2021 ▸; Werner *et al.*, 2014 ▸, 2015 ▸; Suckert *et al.*, 2016 ▸; Wellm *et al.*, 2020 ▸). In this context, urotropine as a coligand was of inter­est because this ligand is able to form networks (Czubacka *et al.*, 2012 ▸; Li *et al.*, 2012 ▸), is magnetically silent and one compound with cadmium had already been reported in which the metal cations are linked by the anionic ligands into chains (Bai *et al.*, 2009 ▸). 
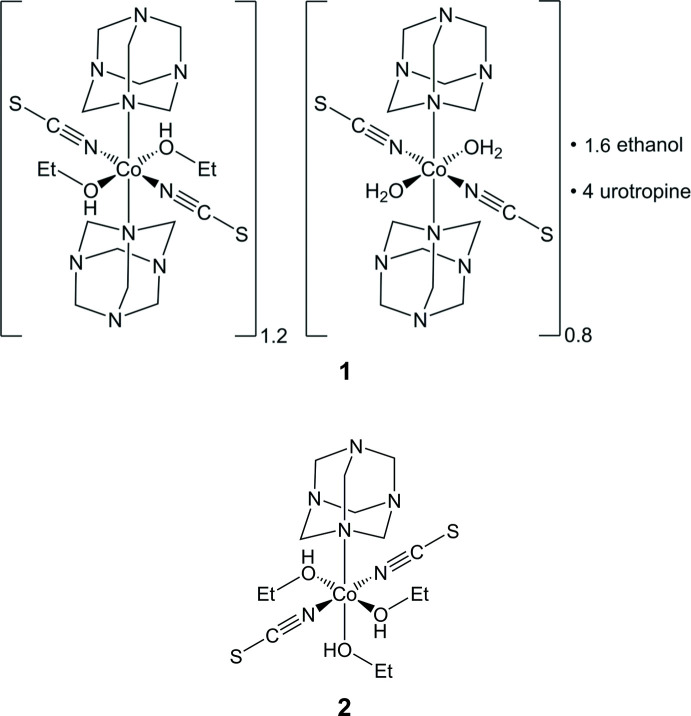


However, for the preparation of the two compounds mentioned above, cobalt thio­cyanate was reacted with urotropine in ethanol and X-ray powder measurements show that none of these compounds can be prepared as a pure crystalline phase. Either the desired compounds were obtained as the minor phase or the experimental powder patterns were completely different from the calculated one. These investigations indicate that additional compounds are present and that the desired compounds are not very stable and transform in solution. Therefore, additional crystallization experiments were performed, which lead to the formation of single crystals of two new compounds that were identified by single crystal X-ray diffraction. Even these compounds contain ethanol as a ligand but in one compound one coord­ination site is simultaneously occupied by ethanol and water, which might originate from some residual water in the solvent used in the synthesis, whereas in the second compound the cobalt cations are coordinated by only one urotropine and three ethanol ligands. All this indicates that, for this system, different species are in equilibrium in solution and some phase crystallizes, presumably by kinetic control, which means that the synthesis is difficult to control.

## Structural commentary

**Figure 1:**
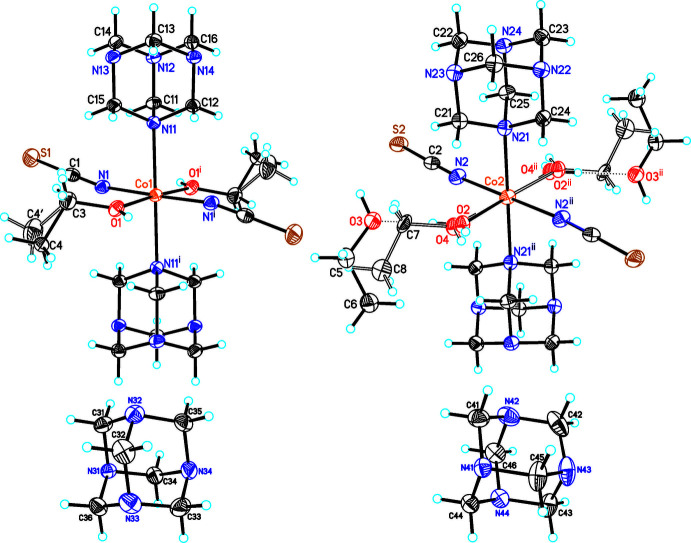
Crystal structure of compound **1** with labelling and displacement ellipsoids drawn at the 50% probability level. Symmetry code for the generation of equivalent atoms: (i) −*x* + 1, −*y* + 1, −*z* + 2; (ii) −*x* + 2, −*y* + 1, −*z* + 1.

The asymmetric unit of compound **1** consists of two crystallographically independent Co cations that are located on centres of inversion as well as two thio­cyanate anions, four urotropine ligands, three ethanol and one water mol­ecule that occupy general positions (Fig. 1 ▸). One of the cobalt cations (Co1) is sixfold coordinated to two terminal N-bonded thio­cyanate anions, two urotropine ligands and two ethanol mol­ecules into discrete complexes (Fig. 1 ▸, top left). The methyl carbon atom of these ethanol mol­ecules is disordered in two positions and was refined using a split model. The second cobalt cation is also sixfold coordinated, forming discrete complexes, to two terminal N-bonded thio­cyanate anions, two urotropine ligands and two oxygen atoms, but the latter positions are mixed occupied by water and ethanol in a ratio of 8:2, leading to an overall composition for **1** of [Co(NCS)_2_(urotropine)_2_(ethanol)_1.2_(H_2_O)_0.8_·1.6ethanol·4urotropine. In the case where it is occupied by water, an ethanol mol­ecule is hydrogen bonded to this water mol­ecule; if it is occupied by ethanol, this ethanol solvate mol­ecule is not present (Fig. 1 ▸, top right). The position of the disordered O atoms of the water and ethanol mol­ecule was resolved and all O—H H atoms were clearly located in the difference map and refined isotropically with reasonable displacement parameters, using restraints for the O—H distances (see *Refinement*). The Co—N bond lengths to the thio­canate anions are similar in both complexes, which is also valid for the bond length to the urotropine ligands (Table 1 ▸). In contrast, the Co—O bond length to the water mol­ecule is shorter than those to the ethanol mol­ecules (Table 1 ▸), even if there might be some uncertainty in the distances because of the disorder.

**Figure 2:**
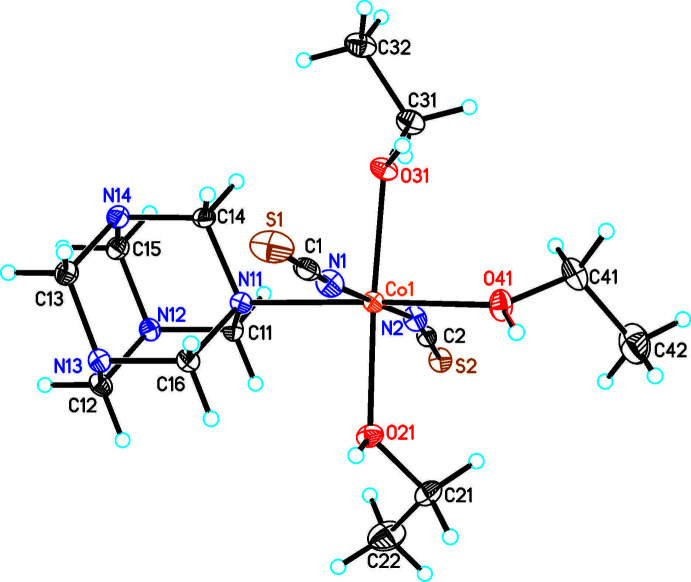
Crystal structure of compound **2** with labelling and displacement ellipsoids drawn at the 50% probability level.

The asymmetric unit of compound **2** consists of one crystallographically independent cobalt cation, one urotropine ligand and three ethanol mol­ecules, all of them located in general positions (Fig. 2 ▸). In this compound the cobalt cations are sixfold coordinated to two terminal N-bonded thio­cyanate anions, one urotropine ligand and three ethanol mol­ecules. The Co—N and Co—O bond lengths are comparable to those in compound **1** and to similar ethanol complexes retrieved from the literature (Krebs *et al.*, 2021*a*, Table 2 ▸). From the angles around the Co cations, it is obvious that in all compounds the octa­hedra are slightly distorted (see supporting information). It is noted that compound **2** completes the series of Co(NCS)_2_-urotropine compounds with ethanol as an additional ligand, because in this compound the cobalt cations are coordinated to one urotropine and three ethanol ligands, whereas in the other compounds reported recently the cobalt cations are either coordinated to two urotropine and two ethanol ligands or to four ethanol ligands (Krebs *et al.*, 2021*a*).

## Supra­molecular features

**Figure 3:**
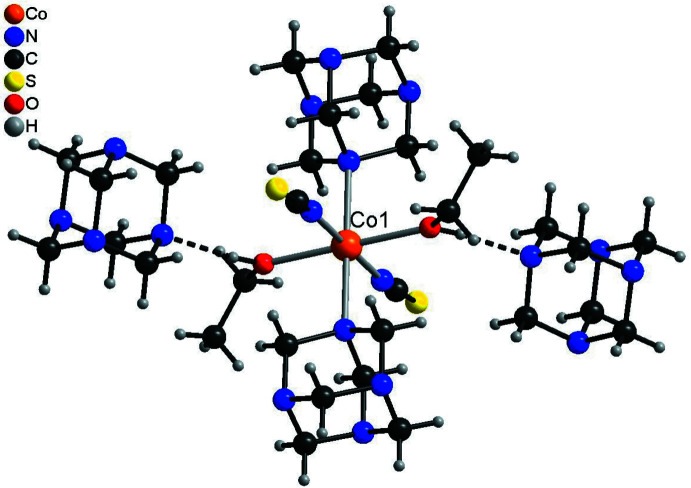
View of the discrete complex in compound **1** built up of Co1, which is connected to two urotropine solvate mol­ecules *via* inter­molecular O—H⋯N hydrogen bonding (shown as dashed lines).

**Figure 4:**
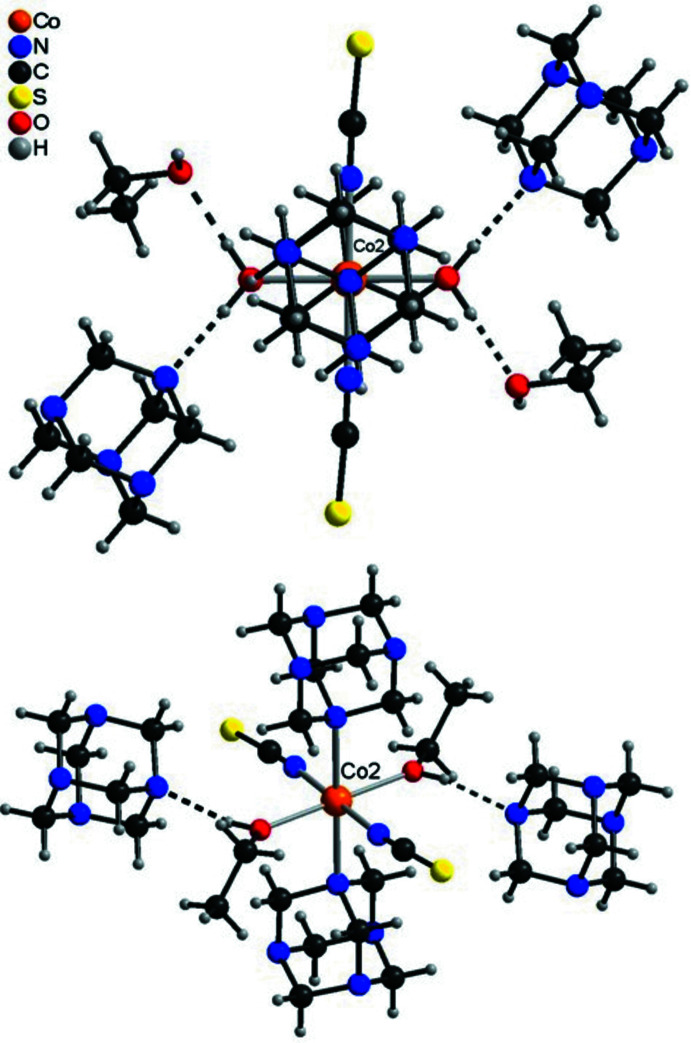
View of the two different coordinations of Co2 in compound **1** with H_2_O (top) and ethanol (bottom) with inter­molecular hydrogen bonding shown as dashed lines.

**Figure 5:**
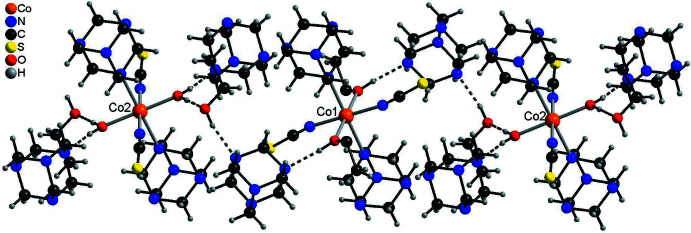
Part of the crystal structure of compound **1** showing the connection of the discrete complexes by the urotropine solvate mol­ecules *via* inter­molecular O—H⋯N hydrogen bonding (shown as dashed lines).

**Figure 6:**
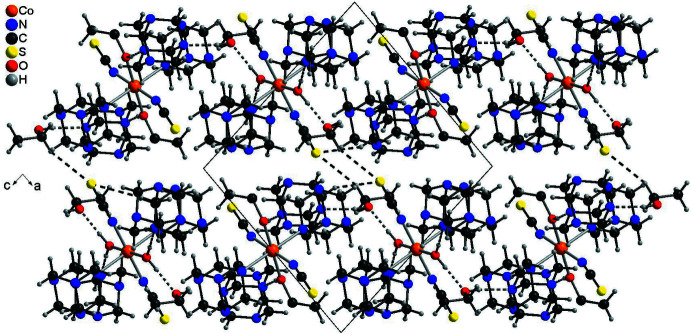
Crystal structure of compound **1** with a view along the crystallographic *b* axis and inter­molecular hydrogen bonding shown as dashed lines.

In the crystal structure of the title compound, extensive hydrogen bonding is observed (Table 3 ▸). The discrete complex around Co1 is linked to two urotropine solvate mol­ecules *via* inter­molecular O—H⋯N hydrogen bonding (Fig. 3 ▸ and Table 3 ▸). For the Co2 complex, two different surroundings are observed. In the case where this cation is coordinated to water, this water mol­ecule is hydrogen bonded to two urotropine ligands and two ethanol mol­ecules (Fig. 4 ▸, top and Table 3 ▸). There are two additional C—H⋯S hydrogen bonds, which are not shown for clarity. In the case where Co2 is coordinated to EtOH, the solvate ethanol mol­ecule is not present and the surrounding is similar to that around Co1 with only hydrogen bonding to two urotropine ligands (compare Fig. 3 ▸ and Fig. 4 ▸, bottom). Both crystallographically independent complexes are linked into chains *via* inter­molecular O—H⋯O and O—H⋯N hydrogen bonding (Fig. 5 ▸). The chains are further connected into layers by inter­molecular C—H⋯O and C—H⋯N inter­actions. These layers are stacked onto each other and are linked by inter­molecular centrosymmetric pairs of C—H⋯S hydrogen bonds, in which only the discrete complex built up of Co2 is involved (Fig. 6 ▸ and Table 3 ▸).

**Figure 7:**
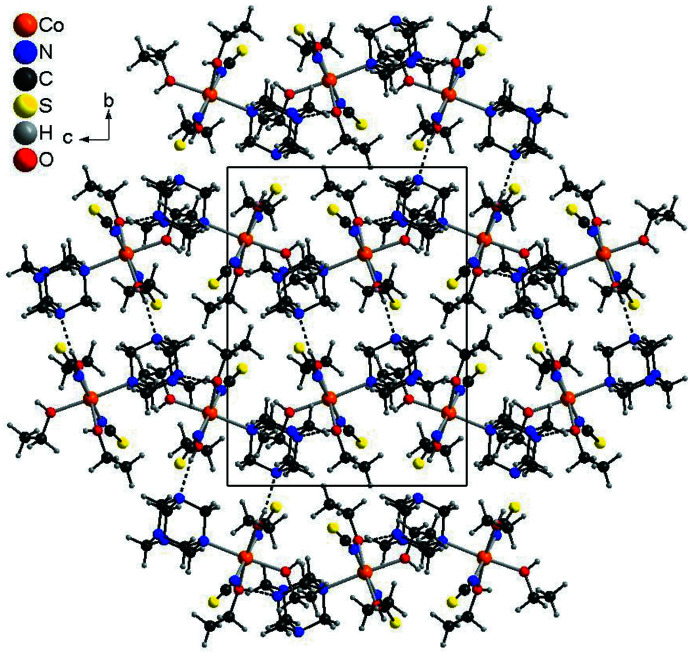
Crystal structure of compound **2** with a view along the crystallographic *a* axis and inter­molecular O—H⋯N hydrogen bonding shown as dashed lines.

In the crystal structure of compound **2**, the discrete complexes are linked by strong inter­molecular O—H⋯N hydrogen bonding between two of the three O—H hydrogen atoms of the ethanol ligands and two urotropine N atoms into layers that are parallel to the *bc* plane (Fig. 7 ▸ and Table 4 ▸). These layers are further linked by inter­molecular O—H⋯S and C—H⋯S hydrogen bonding into a three-dimensional network (Table 4 ▸). Some of the O—H⋯S and C—H⋯S angles are close to linearity, indicating that these are relatively strong inter­actions (Table 4 ▸).

## Database survey

In the Cambridge Structure Database (CSD version 5.42, last update November 2020; Groom *et al.*, 2016 ▸) there are already several structures reported that contain cobalt thio­cyanate and urotropine as a ligand, but only one of them contains additional ethanol (Krebs *et al.*, 2021*a*). Most of them contain water as a ligand or solvate mol­ecule. In [Co(NCS)_2_(H_2_O)_4_]·2urotropine (Refcode: XILXOG; Li *et al.*, 2007 ▸), the cobalt cations are octa­hedrally coordinated by two thio­cyanate anions and four water ligands with two additional urotropine ligands acting as solvate mol­ecules. [Co(NCS)_2_(urotrop­ine)_2_(H_2_O)_2_][Co(NCS)_2_(H_2_O)_4_]·2H_2_O (Refcode: MOTNIS; Liu *et al.*, 2002 ▸, MOTNIS01; Zhang *et al.*, 1999 ▸, MOTNIS02; Chakraborty *et al.*, 2006 ▸, MOTNIS03; Lu *et al.*, 2010 ▸) consists of two crystallographically independent discrete complexes in which the cobalt cations are coordinated by two terminal N-bonded thio­cyanate anions and four water or two water and two urotropine ligands with additional water as solvate mol­ecules. There is also one complex with water and methanol as ligands with the composition [Co(NCS)_2_(urotropine)(CH_3_OH)_2_(H_2_O)] (Refcode: POFGAT; Shang *et al.*, 2008 ▸), in which the cobalt cations are octa­hedrally coordinated by the N atoms of two thio­cyanate anions, two methanol, one water and one urotropine ligand. Moreover, a compound with the composition [Co(NCS)_2_(urotropine)_2_(CH_3_CN)_2_] that also consists of discrete complexes has been reported (Krebs *et al.*, 2021 ▸). It is noted that even with other metal cations only discrete complexes are reported, such as, for example, with nickel (Refcode: XILROA; Bai *et al.*, 2007 ▸, XILROA01; Lu *et al.*, 2010 ▸), or zinc (Refcode: SIMXIY; Kruszynski *et al.*, 2018). Finally, a crystal structure is reported with cadmium in which the Cd cations are linked by pairs of thio­cyanate anions into chains, which are further linked by the urotropine ligand (Refcode: DOZZOI; Bai *et al.*, 2009 ▸).

## Synthesis and crystallization

**Synthesis** Co(NCS)_2_ and urotropine were purchased from Merck. All chemicals were used without further purification.

Crystals of compound **1** suitable for single-crystal X-ray diffraction were obtained after one day by the reaction of 0.15 mmol of Co(NCS)_2_ (26.3 mg) with 0.60 mmol of urotropine (84.1 mg) in 1.0 mL of ethanol at room temperature. The reaction of 0.15 mmol of Co(NCS)_2_ (26.3 mg) with 0.15 mmol of urotropine (21.0 mg) in 2.0 mL of ethanol at room temperature led to the formation of single crystals of compound **2**.

The data collection for single-crystal structure analysis was performed using an XtaLAB Synergy, Dualflex, HyPix diffractometer from Rigaku with Cu *K*α radiation.

## Refinement

Crystal data, data collection and structure refinement details are summarized in Table 5 ▸. All non-hydrogen atoms were refined anisotropically. The C—H hydrogen atoms were located in the difference map but positioned with idealized geometry (methyl H atoms allowed to rotate but not to tip) and were refined isotropically with *U*_iso_(H) = 1.2*U*_eq_(C) (1.5 for methyl H atoms) using a riding model. The O—H hydrogen atoms were located in the difference map and were refined with restraints for the O—H distance (DFIX) and varying isotropic displacement parameters in compound **1**, whereas in compound **2** they were positioned with idealized geometry allowed to rotate but not to tip and were refined isotropically with *U*_iso_(H) = 1.5*U*_eq_(O) using a riding model. In compound **1**, the methyl group of the EtOH mol­ecule coordinated to Co1 is disordered and was refined using a split model. In this compound, Co2 is either coordinated to water or to EtOH. In this case the O atoms occupy nearly the same crystallographic positions but finally both O atoms can be refined separately with anisotropic displacement parameters. In the case where Co2 is coordinated to water, it is hydrogen bonded to one EtOH solvate mol­ecule. If Co2 is coordinated to EtOH, the position of the EtOH solvate mol­ecule cannot be occupied. Therefore, the site occupation factor (sof) of the EtOH solvate mol­ecule must be identical to that of the coordinated water mol­ecule. In the beginning the sof was refined, leading to values close to 0.8 for the water and 0.2 for the coordinated EtOH mol­ecule but in the final refinements it was fixed at 0.8 and 0.2. The H-atom positions of both, water and EtOH, were clearly located and were refined with restraints and varying isotropic displacement parameters. This leads to comparable and reasonable values for the O—H distances as well as for the isotropic displacement parameters of the O—H hydrogen atoms.

## Supplementary Material

Crystal structure: contains datablock(s) 1, 2. DOI: 10.1107/S2056989022001037/tx2047sup1.cif

Structure factors: contains datablock(s) 1. DOI: 10.1107/S2056989022001037/tx20471sup2.hkl

Structure factors: contains datablock(s) 2. DOI: 10.1107/S2056989022001037/tx20472sup3.hkl

CCDC references: 2145766, 2145767

Additional supporting information:  crystallographic information; 3D view; checkCIF report

## Figures and Tables

**Table 1:** Selected bond lengths (Å) for **1** [Chem scheme1]

Co1—N1	2.0590 (16)	Co2—O2	2.029 (6)
Co1—O1	2.1388 (13)	Co2—O4	2.21 (3)
Co1—N11	2.2834 (15)	Co2—N21	2.2788 (16)
Co2—N2	2.0812 (16)		

**Table 2:** Selected bond lengths (Å) for **2** [Chem scheme1]

Co1—N2	2.0615 (11)	Co1—O31	2.1157 (9)
Co1—N1	2.0624 (11)	Co1—O21	2.1314 (9)
Co1—O41	2.1021 (10)	Co1—N11	2.2489 (11)

**Table 3:** Hydrogen-bond geometry (Å, °) for **1** [Chem scheme1]

*D*—H⋯*A*	*D*—H	H⋯*A*	*D*⋯*A*	*D*—H⋯*A*
O1—H1⋯N31	0.88 (2)	1.92 (2)	2.793 (2)	170 (3)
C4′—H4′*A*⋯N43^i^	0.96	2.50	3.243 (14)	134
C4′—H4′*C*⋯N44^ii^	0.96	2.38	3.161 (10)	138
O2—H2*A*⋯N41	0.87 (2)	1.88 (2)	2.743 (7)	173 (7)
O2—H2*B*⋯O3	0.87 (2)	1.80 (2)	2.665 (4)	177 (5)
C5—H5*A*⋯S2^iii^	0.97	3.02	3.925 (3)	156
O3—H3⋯N34	0.87 (2)	1.97 (2)	2.821 (3)	167 (4)
O4—H4⋯N41	0.87 (2)	1.94 (5)	2.81 (3)	170 (19)
C11—H11*A*⋯O1^ii^	0.97	2.49	3.058 (2)	117
C11—H11*B*⋯N1	0.97	2.67	3.213 (2)	116
C12—H12*B*⋯N44	0.97	2.64	3.423 (3)	138
C13—H13*A*⋯N13^iv^	0.97	2.70	3.563 (2)	149
C13—H13*B*⋯S2^iii^	0.97	2.95	3.7150 (19)	136
C14—H14*A*⋯S2^v^	0.97	2.93	3.840 (2)	156
C15—H15*B*⋯O1	0.97	2.61	3.118 (2)	113
C22—H22*B*⋯N12^vi^	0.97	2.58	3.448 (2)	149
C25—H25*A*⋯O2^vii^	0.97	2.50	3.026 (7)	114
C25—H25*A*⋯O4^vii^	0.97	2.49	3.08 (3)	119
C25—H25*B*⋯N2	0.97	2.61	3.202 (3)	119
C26—H26*A*⋯S1^ii^	0.97	2.98	3.655 (2)	128
C26—H26*B*⋯N22^viii^	0.97	2.69	3.581 (3)	152
C33—H33*A*⋯N23	0.97	2.66	3.431 (3)	137
C45—H45*A*⋯S2^vii^	0.97	3.01	3.959 (3)	165

Symmetry codes: (i) 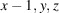 ; (ii) 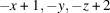 ; (iii) 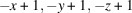 ; (iv) 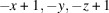 ; (v) 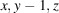 ; (vi) 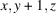 ; (vii) 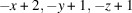 ; (viii) 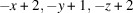 .

**Table 4:** Hydrogen-bond geometry (Å, °) for **2** [Chem scheme1]

*D*—H⋯*A*	*D*—H	H⋯*A*	*D*⋯*A*	*D*—H⋯*A*
C12—H12*A*⋯S2^i^	0.99	2.87	3.6586 (13)	137
C12—H12*B*⋯S1^ii^	0.99	2.92	3.8813 (13)	164
C15—H15*A*⋯S1^iii^	0.99	2.99	3.9387 (13)	161
C15—H15*B*⋯S2^iv^	0.99	2.94	3.7110 (13)	135
C16—H16*A*⋯O21	0.99	2.54	3.1009 (16)	116
C16—H16*B*⋯N1	0.99	2.47	3.1083 (17)	122
O21—H21⋯N13^ii^	0.84	2.03	2.8424 (14)	161
C22—H22*C*⋯S1^v^	0.98	3.02	3.9559 (16)	161
O31—H31⋯N12^vi^	0.84	1.96	2.7969 (14)	172
O41—H41⋯S2^vi^	0.84	2.37	3.2080 (10)	174

Symmetry codes: (i) 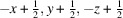 ; (ii) 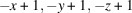 ; (iii) 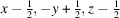 ; (iv) 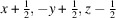 ; (v) 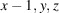 ; (vi) 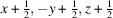 .

**Table 5:** Experimental details

	**1**	**2**
Crystal data		
Chemical formula	[Co(NCS)_2_(C_6_H_12_N_4_)_2_(C_2_H_6_O)_2_]_1.2_·[Co(NCS)_2_(C_6_H_12_N_4_)_2_(H_2_O)_2_]_0.8_·1.6C_2_H_6_O·4C_6_H_12_N_4_	[Co(NCS)_2_(C_6_H_12_N_4_)(C_2_H_6_O)_3_]
*M*_r_	1684.84	453.49
Crystal system, space group	Triclinic, *P* 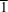	Monoclinic, *P*2_1_/*n*
Temperature (K)	100	100
*a*, *b*, *c* (Å)	12.1536 (2), 12.9256 (3), 12.9374 (3)	11.1463 (1), 15.7705 (1), 12.1824 (1)
α, β, γ (°)	76.629 (2), 80.395 (2), 80.578 (2)	90, 103.886 (1), 90
*V* (Å^3^)	1932.91 (7)	2078.87 (3)
*Z*	1	4
Radiation type	Cu *K*α	Cu *K*α
μ (mm^−1^)	4.97	8.58
Crystal size (mm)	0.16 × 0.12 × 0.08	0.2 × 0.18 × 0.03

Data collection		
Diffractometer	XtaLAB Synergy, Dualflex, HyPix	XtaLAB Synergy, Dualflex, HyPix
Absorption correction	Multi-scan (*CrysAlis PRO*; Rigaku OD, 2021 ▸)	Multi-scan (*CrysAlis PRO*; Rigaku OD, 2021 ▸)
*T*_min_, *T*_max_	0.693, 1.000	0.427, 1.000
No. of measured, independent and observed [*I* > 2σ(*I*)] reflections	25821, 8226, 7777	29441, 4431, 4373
*R*_int_	0.024	0.027
(sin θ/λ)_max_ (Å^−1^)	0.639	0.635

Refinement		
*R*[*F* ^2^ > 2σ(*F* ^2^)], *wR*(*F* ^2^), *S*	0.040, 0.103, 1.09	0.025, 0.068, 1.08
No. of reflections	8226	4431
No. of parameters	545	242
No. of restraints	10	1
H-atom treatment	H atoms treated by a mixture of independent and constrained refinement	H-atom parameters constrained
Δρ_max_, Δρ_min_ (e Å^−3^)	0.82, −0.69	0.32, −0.31

Computer programs: *CrysAlis PRO* (Rigaku OD, 2021 ▸), *SHELXT2014/4* and *SHELXT2014/5* (Sheldrick, 2015*a* ▸), *SHELXL2016/6* (Sheldrick, 2015*b* ▸), *DIAMOND* (Brandenburg & Putz, 1999 ▸), *OLEX2* (Dolomanov *et al.*, 2009 ▸) and *publCIF* (Westrip, 2010 ▸).

## supplementary crystallographic information

### Bis(ethanol-κO)bis(hexamethylenetetramine-κN)bis(thiocyanato-κN)cobalt(II)–diaqua-κ2O-bis(hexamethylenetetramine-κN)bis(thiocyanato-κN)cobalt(II)–ethanol–hexamethylenetetramine (1.2/0.8/1.6/4) (1) Crystal data

**Table:**

[Co(NCS)_2_(C_6_H_12_N_4_)_2_(C_2_H_6_O)_2_]_1.2_·[Co(NCS)_2_(C_6_H_12_N_4_)_2_(H_2_O)_2_]_0.8_·1.6C_2_H_6_O·4C_6_H_12_N_4_	*Z* = 1
*M**_r_* = 1684.84	*F*(000) = 898
Triclinic, *P*1	*D*_x_ = 1.447 Mg m^−^^3^
*a* = 12.1536 (2) Å	Cu *K*α radiation, λ = 1.54178 Å
*b* = 12.9256 (3) Å	Cell parameters from 18138 reflections
*c* = 12.9374 (3) Å	θ = 3.7–79.3°
α = 76.629 (2)°	µ = 4.97 mm^−^^1^
β = 80.395 (2)°	*T* = 100 K
γ = 80.578 (2)°	Plate, light colourless
*V* = 1932.91 (7) Å^3^	0.16 × 0.12 × 0.08 mm

### Bis(ethanol-κO)bis(hexamethylenetetramine-κN)bis(thiocyanato-κN)cobalt(II)–diaqua-κ2O-bis(hexamethylenetetramine-κN)bis(thiocyanato-κN)cobalt(II)–ethanol–hexamethylenetetramine (1.2/0.8/1.6/4) (1) Data collection

**Table:**

XtaLAB Synergy, Dualflex, HyPix diffractometer	8226 independent reflections
Radiation source: micro-focus sealed X-ray tube, PhotonJet (Cu) X-ray Source	7777 reflections with *I* > 2σ(*I*)
Mirror monochromator	*R*_int_ = 0.024
Detector resolution: 10.0000 pixels mm^-1^	θ_max_ = 80.1°, θ_min_ = 3.5°
ω scans	*h* = −15→15
Absorption correction: multi-scan (CrysAlisPro; Rigaku OD, 2021)	*k* = −16→13
*T*_min_ = 0.693, *T*_max_ = 1.000	*l* = −16→16
25821 measured reflections	

### Bis(ethanol-κO)bis(hexamethylenetetramine-κN)bis(thiocyanato-κN)cobalt(II)–diaqua-κ2O-bis(hexamethylenetetramine-κN)bis(thiocyanato-κN)cobalt(II)–ethanol–hexamethylenetetramine (1.2/0.8/1.6/4) (1) Refinement

**Table:**

Refinement on *F*^2^	Hydrogen site location: mixed
Least-squares matrix: full	H atoms treated by a mixture of independent and constrained refinement
*R*[*F*^2^ > 2σ(*F*^2^)] = 0.040	*w* = 1/[σ^2^(*F*_o_^2^) + (0.0432*P*)^2^ + 1.7899*P*] where *P* = (*F*_o_^2^ + 2*F*_c_^2^)/3
*wR*(*F*^2^) = 0.103	(Δ/σ)_max_ = 0.001
*S* = 1.09	Δρ_max_ = 0.82 e Å^−^^3^
8226 reflections	Δρ_min_ = −0.69 e Å^−^^3^
545 parameters	Extinction correction: SHELXL2016/6 (Sheldrick 2015b), Fc^*^=kFc[1+0.001xFc^2^λ^3^/sin(2θ)]^-1/4^
10 restraints	Extinction coefficient: 0.00080 (10)
Primary atom site location: dual	

### Bis(ethanol-κO)bis(hexamethylenetetramine-κN)bis(thiocyanato-κN)cobalt(II)–diaqua-κ2O-bis(hexamethylenetetramine-κN)bis(thiocyanato-κN)cobalt(II)–ethanol–hexamethylenetetramine (1.2/0.8/1.6/4) (1) Special details

**Table:**

Geometry. All esds (except the esd in the dihedral angle between two l.s. planes) are estimated using the full covariance matrix. The cell esds are taken into account individually in the estimation of esds in distances, angles and torsion angles; correlations between esds in cell parameters are only used when they are defined by crystal symmetry. An approximate (isotropic) treatment of cell esds is used for estimating esds involving l.s. planes.

### Bis(ethanol-κO)bis(hexamethylenetetramine-κN)bis(thiocyanato-κN)cobalt(II)–diaqua-κ2O-bis(hexamethylenetetramine-κN)bis(thiocyanato-κN)cobalt(II)–ethanol–hexamethylenetetramine (1.2/0.8/1.6/4) (1) Fractional atomic coordinates and isotropic or equivalent isotropic displacement parameters (Å^2^)

**Table:**

	*x*	*y*	*z*	*U*_iso_*/*U*_eq_	Occ. (<1)
Co1	0.500000	0.000000	1.000000	0.01667 (10)	
Co2	1.000000	0.500000	0.500000	0.01929 (11)	
N1	0.38177 (14)	−0.10448 (13)	1.03166 (13)	0.0209 (3)	
C1	0.31474 (16)	−0.15722 (15)	1.02515 (15)	0.0219 (4)	
S1	0.22329 (5)	−0.23250 (5)	1.01546 (5)	0.03988 (15)	
N2	0.85107 (14)	0.59836 (13)	0.46671 (13)	0.0238 (3)	
C2	0.76974 (17)	0.65684 (15)	0.45319 (15)	0.0226 (4)	
S2	0.65758 (5)	0.74450 (5)	0.43091 (5)	0.03506 (14)	
O1	0.37719 (11)	0.13161 (11)	0.94616 (11)	0.0216 (3)	
H1	0.392 (3)	0.1982 (16)	0.927 (3)	0.060 (10)*	
C3	0.26483 (18)	0.13151 (18)	0.91942 (18)	0.0294 (4)	
H3AA	0.250342	0.057923	0.930034	0.044*	0.8
H3AB	0.262932	0.164417	0.844216	0.044*	0.8
H3BC	0.250431	0.196444	0.865692	0.044*	0.2
H3BD	0.271029	0.072225	0.883622	0.044*	0.2
C4	0.1725 (2)	0.1909 (2)	0.98618 (19)	0.0200 (4)	0.8
H4A	0.100686	0.185071	0.968034	0.030*	0.8
H4B	0.183149	0.265097	0.971950	0.030*	0.8
H4C	0.175324	0.160103	1.060858	0.030*	0.8
C4'	0.1630 (6)	0.1249 (14)	0.9957 (8)	0.047 (3)	0.2
H4'A	0.104113	0.109715	0.962611	0.071*	0.2
H4'B	0.140682	0.191965	1.018112	0.071*	0.2
H4'C	0.176793	0.068708	1.056935	0.071*	0.2
O2	0.9172 (3)	0.3693 (6)	0.5467 (6)	0.0215 (6)	0.8
H2A	0.939 (5)	0.308 (3)	0.588 (5)	0.07 (3)*	0.8
H2B	0.8447 (17)	0.372 (4)	0.548 (4)	0.077 (18)*	0.8
O3	0.69673 (15)	0.37997 (14)	0.54414 (15)	0.0275 (4)	0.8
C5	0.6645 (2)	0.2810 (2)	0.5350 (2)	0.0267 (5)	0.8
H5A	0.586260	0.291950	0.523215	0.032*	0.8
H5B	0.672275	0.228069	0.600949	0.032*	0.8
C6	0.7380 (2)	0.2411 (2)	0.4427 (2)	0.0298 (5)	0.8
H6A	0.731836	0.294723	0.378049	0.045*	0.8
H6B	0.714164	0.176471	0.434837	0.045*	0.8
H6C	0.814805	0.226809	0.456517	0.045*	0.8
H3	0.656 (3)	0.406 (3)	0.597 (2)	0.054 (11)*	0.8
O4	0.8970 (16)	0.367 (3)	0.543 (3)	0.0215 (6)	0.2
C7	0.7799 (8)	0.3562 (8)	0.5603 (9)	0.026 (2)	0.2
H7A	0.736856	0.426993	0.546472	0.032*	0.2
H7B	0.759133	0.323060	0.634838	0.032*	0.2
C8	0.7500 (9)	0.2899 (8)	0.4899 (8)	0.030 (2)	0.2
H8A	0.762768	0.326382	0.416213	0.046*	0.2
H8B	0.672194	0.279629	0.508926	0.046*	0.2
H8C	0.796095	0.221486	0.499688	0.046*	0.2
H4	0.930 (14)	0.306 (9)	0.574 (17)	0.02 (5)*	0.2
N11	0.53970 (13)	−0.04050 (12)	0.83394 (12)	0.0169 (3)	
N12	0.59878 (13)	−0.18558 (13)	0.73316 (12)	0.0199 (3)	
N13	0.46941 (13)	−0.02933 (13)	0.66262 (12)	0.0201 (3)	
N14	0.66683 (13)	−0.01109 (12)	0.66326 (12)	0.0189 (3)	
C11	0.57045 (16)	−0.15859 (14)	0.83915 (14)	0.0193 (4)	
H11A	0.634285	−0.184309	0.878516	0.023*	
H11B	0.507809	−0.195364	0.878363	0.023*	
C12	0.63690 (15)	0.01248 (15)	0.77084 (14)	0.0189 (4)	
H12A	0.618761	0.089443	0.764292	0.023*	
H12B	0.701533	−0.011012	0.809414	0.023*	
C13	0.56767 (16)	0.02352 (15)	0.60617 (15)	0.0213 (4)	
H13A	0.586413	0.007641	0.535122	0.026*	
H13B	0.548205	0.100566	0.597793	0.026*	
C14	0.50120 (16)	−0.14575 (16)	0.67457 (15)	0.0220 (4)	
H14A	0.518813	−0.163034	0.604076	0.026*	
H14B	0.437746	−0.181983	0.712554	0.026*	
C15	0.44349 (16)	−0.00438 (15)	0.76983 (15)	0.0205 (4)	
H15A	0.378951	−0.038674	0.808307	0.025*	
H15B	0.423192	0.072471	0.762594	0.025*	
C16	0.69351 (16)	−0.12818 (15)	0.67461 (15)	0.0208 (4)	
H16A	0.758527	−0.153184	0.712455	0.025*	
H16B	0.713010	−0.144866	0.603934	0.025*	
N21	0.96389 (13)	0.53277 (12)	0.66888 (12)	0.0185 (3)	
N22	1.03238 (14)	0.51130 (13)	0.84249 (13)	0.0209 (3)	
N23	0.83260 (14)	0.50888 (13)	0.83676 (13)	0.0211 (3)	
N24	0.91408 (14)	0.67678 (13)	0.77182 (13)	0.0207 (3)	
C21	0.86158 (16)	0.48569 (15)	0.72915 (15)	0.0206 (4)	
H21A	0.798587	0.514069	0.689148	0.025*	
H21B	0.874416	0.408596	0.735116	0.025*	
C22	0.81436 (16)	0.62564 (15)	0.82683 (16)	0.0221 (4)	
H22A	0.794956	0.641618	0.897807	0.027*	
H22B	0.751325	0.655797	0.787314	0.027*	
C23	1.00907 (16)	0.62886 (15)	0.83182 (16)	0.0226 (4)	
H23A	0.992555	0.645241	0.902716	0.027*	
H23B	1.075775	0.660737	0.795445	0.027*	
C24	1.05778 (16)	0.48863 (15)	0.73384 (15)	0.0201 (4)	
H24A	1.073004	0.411640	0.739378	0.024*	
H24B	1.125119	0.519334	0.697337	0.024*	
C25	0.94127 (16)	0.65112 (14)	0.66518 (15)	0.0205 (4)	
H25A	1.007066	0.683951	0.627731	0.025*	
H25B	0.879088	0.681840	0.624686	0.025*	
C26	0.92924 (16)	0.46570 (15)	0.89559 (15)	0.0217 (4)	
H26A	0.942690	0.388452	0.902783	0.026*	
H26B	0.911220	0.480513	0.967089	0.026*	
N31	0.41819 (13)	0.34324 (13)	0.86223 (13)	0.0213 (3)	
N32	0.34067 (15)	0.51211 (14)	0.75097 (14)	0.0266 (4)	
N33	0.47210 (17)	0.51353 (15)	0.87450 (15)	0.0325 (4)	
N34	0.53923 (15)	0.44041 (15)	0.71314 (14)	0.0289 (4)	
C31	0.31950 (17)	0.40495 (16)	0.81169 (17)	0.0266 (4)	
H31A	0.299473	0.365307	0.763932	0.032*	
H31B	0.255972	0.411911	0.867126	0.032*	
C32	0.3720 (2)	0.56912 (17)	0.82560 (18)	0.0329 (5)	
H32A	0.386349	0.640567	0.787339	0.040*	
H32B	0.309371	0.576543	0.881808	0.040*	
C33	0.56536 (19)	0.50044 (19)	0.78861 (19)	0.0349 (5)	
H33A	0.631573	0.462773	0.820177	0.042*	
H33B	0.582728	0.570673	0.749263	0.042*	
C34	0.51269 (18)	0.33500 (16)	0.77563 (16)	0.0272 (4)	
H34A	0.578777	0.295933	0.806522	0.033*	
H34B	0.494052	0.294447	0.727982	0.033*	
C35	0.43694 (18)	0.49921 (17)	0.66807 (17)	0.0291 (4)	
H35A	0.452907	0.569471	0.627554	0.035*	
H35B	0.417450	0.460785	0.618972	0.035*	
C36	0.44831 (19)	0.40664 (17)	0.93320 (16)	0.0275 (4)	
H36A	0.386711	0.413627	0.990439	0.033*	
H36B	0.514013	0.368715	0.965490	0.033*	
N41	0.98222 (14)	0.16814 (13)	0.66156 (13)	0.0236 (3)	
N42	1.07891 (16)	−0.00576 (15)	0.62924 (17)	0.0350 (4)	
N43	1.09861 (17)	0.05352 (15)	0.79123 (17)	0.0379 (5)	
N44	0.92004 (15)	0.00149 (14)	0.77178 (14)	0.0276 (4)	
C41	1.04120 (19)	0.10589 (18)	0.58097 (18)	0.0316 (5)	
H41A	0.990963	0.107214	0.529629	0.038*	
H41B	1.105888	0.139850	0.542356	0.038*	
C42	1.15409 (19)	−0.00508 (19)	0.7069 (2)	0.0420 (6)	
H42A	1.219661	0.028026	0.669308	0.050*	
H42B	1.179667	−0.078443	0.740249	0.050*	
C43	0.9988 (2)	0.00220 (19)	0.84575 (19)	0.0358 (5)	
H43A	1.022883	−0.071033	0.880625	0.043*	
H43B	0.960481	0.040129	0.900804	0.043*	
C44	0.88530 (17)	0.11291 (16)	0.71995 (17)	0.0260 (4)	
H44A	0.845718	0.151596	0.773986	0.031*	
H44B	0.833555	0.113901	0.670081	0.031*	
C45	1.0595 (2)	0.16406 (17)	0.7394 (2)	0.0348 (5)	
H45A	1.124033	0.199417	0.702869	0.042*	
H45B	1.021402	0.202857	0.793862	0.042*	
C46	0.98007 (19)	−0.05437 (17)	0.68850 (19)	0.0326 (5)	
H46A	0.928905	−0.054578	0.638482	0.039*	
H46B	1.003704	−0.128358	0.721415	0.039*	

### Bis(ethanol-κO)bis(hexamethylenetetramine-κN)bis(thiocyanato-κN)cobalt(II)–diaqua-κ2O-bis(hexamethylenetetramine-κN)bis(thiocyanato-κN)cobalt(II)–ethanol–hexamethylenetetramine (1.2/0.8/1.6/4) (1) Atomic displacement parameters (Å^2^)

**Table:**

	*U*^11^	*U*^22^	*U*^33^	*U*^12^	*U*^13^	*U*^23^
Co1	0.0194 (2)	0.0162 (2)	0.0148 (2)	−0.00317 (15)	−0.00286 (15)	−0.00303 (15)
Co2	0.0223 (2)	0.0144 (2)	0.0185 (2)	0.00176 (16)	−0.00397 (16)	−0.00022 (16)
N1	0.0246 (8)	0.0200 (7)	0.0189 (7)	−0.0060 (6)	−0.0018 (6)	−0.0045 (6)
C1	0.0258 (9)	0.0193 (9)	0.0184 (9)	−0.0004 (7)	−0.0006 (7)	−0.0029 (7)
S1	0.0345 (3)	0.0400 (3)	0.0532 (4)	−0.0172 (2)	0.0012 (3)	−0.0224 (3)
N2	0.0247 (8)	0.0206 (8)	0.0232 (8)	0.0042 (6)	−0.0050 (6)	−0.0025 (6)
C2	0.0267 (10)	0.0226 (9)	0.0199 (9)	−0.0029 (8)	−0.0031 (7)	−0.0075 (7)
S2	0.0283 (3)	0.0407 (3)	0.0403 (3)	0.0136 (2)	−0.0156 (2)	−0.0217 (2)
O1	0.0244 (7)	0.0192 (6)	0.0222 (6)	−0.0027 (5)	−0.0082 (5)	−0.0030 (5)
C3	0.0272 (10)	0.0309 (11)	0.0322 (11)	−0.0013 (8)	−0.0083 (8)	−0.0091 (9)
C4	0.0190 (11)	0.0222 (12)	0.0184 (11)	−0.0034 (10)	−0.0006 (8)	−0.0044 (10)
C4'	0.042 (7)	0.059 (10)	0.040 (7)	−0.022 (7)	−0.012 (6)	0.006 (7)
O2	0.0187 (17)	0.0192 (8)	0.0251 (10)	0.0002 (16)	−0.0061 (15)	−0.0012 (7)
O3	0.0245 (10)	0.0260 (9)	0.0324 (10)	−0.0041 (7)	−0.0003 (7)	−0.0087 (7)
C5	0.0244 (13)	0.0246 (12)	0.0298 (13)	−0.0041 (10)	−0.0060 (10)	−0.0004 (10)
C6	0.0291 (13)	0.0293 (13)	0.0308 (14)	−0.0040 (11)	−0.0050 (11)	−0.0052 (11)
O4	0.0187 (17)	0.0192 (8)	0.0251 (10)	0.0002 (16)	−0.0061 (15)	−0.0012 (7)
C7	0.013 (5)	0.028 (5)	0.035 (5)	0.003 (4)	0.002 (4)	−0.008 (4)
C8	0.032 (6)	0.024 (5)	0.028 (5)	−0.001 (4)	−0.006 (4)	0.007 (4)
N11	0.0173 (7)	0.0173 (7)	0.0149 (7)	−0.0003 (6)	−0.0035 (5)	−0.0016 (6)
N12	0.0233 (8)	0.0198 (7)	0.0168 (7)	0.0004 (6)	−0.0046 (6)	−0.0051 (6)
N13	0.0207 (8)	0.0226 (8)	0.0175 (7)	0.0011 (6)	−0.0057 (6)	−0.0060 (6)
N14	0.0203 (7)	0.0204 (8)	0.0154 (7)	−0.0004 (6)	−0.0030 (6)	−0.0041 (6)
C11	0.0247 (9)	0.0165 (8)	0.0163 (8)	−0.0009 (7)	−0.0039 (7)	−0.0033 (7)
C12	0.0204 (9)	0.0208 (9)	0.0152 (8)	−0.0024 (7)	−0.0023 (7)	−0.0033 (7)
C13	0.0233 (9)	0.0225 (9)	0.0165 (8)	0.0017 (7)	−0.0057 (7)	−0.0022 (7)
C14	0.0227 (9)	0.0242 (9)	0.0214 (9)	−0.0016 (7)	−0.0064 (7)	−0.0076 (7)
C15	0.0193 (9)	0.0237 (9)	0.0188 (9)	0.0012 (7)	−0.0044 (7)	−0.0067 (7)
C16	0.0194 (9)	0.0222 (9)	0.0199 (9)	0.0026 (7)	−0.0042 (7)	−0.0053 (7)
N21	0.0185 (7)	0.0153 (7)	0.0200 (7)	−0.0003 (6)	−0.0041 (6)	−0.0004 (6)
N22	0.0226 (8)	0.0189 (7)	0.0209 (8)	0.0001 (6)	−0.0060 (6)	−0.0031 (6)
N23	0.0215 (8)	0.0212 (8)	0.0197 (8)	−0.0026 (6)	−0.0032 (6)	−0.0024 (6)
N24	0.0226 (8)	0.0186 (7)	0.0198 (8)	−0.0002 (6)	−0.0033 (6)	−0.0032 (6)
C21	0.0208 (9)	0.0192 (9)	0.0210 (9)	−0.0024 (7)	−0.0036 (7)	−0.0023 (7)
C22	0.0197 (9)	0.0217 (9)	0.0232 (9)	0.0012 (7)	−0.0032 (7)	−0.0041 (7)
C23	0.0232 (9)	0.0202 (9)	0.0251 (9)	−0.0020 (7)	−0.0073 (7)	−0.0041 (7)
C24	0.0192 (8)	0.0188 (8)	0.0210 (9)	0.0008 (7)	−0.0044 (7)	−0.0028 (7)
C25	0.0246 (9)	0.0154 (8)	0.0197 (9)	−0.0004 (7)	−0.0031 (7)	−0.0014 (7)
C26	0.0239 (9)	0.0197 (9)	0.0196 (9)	−0.0017 (7)	−0.0049 (7)	0.0003 (7)
N31	0.0206 (8)	0.0206 (8)	0.0227 (8)	−0.0029 (6)	−0.0045 (6)	−0.0032 (6)
N32	0.0251 (8)	0.0217 (8)	0.0312 (9)	−0.0001 (7)	−0.0056 (7)	−0.0025 (7)
N33	0.0441 (11)	0.0273 (9)	0.0303 (9)	−0.0127 (8)	−0.0096 (8)	−0.0056 (7)
N34	0.0246 (8)	0.0307 (9)	0.0258 (9)	−0.0009 (7)	−0.0019 (7)	0.0022 (7)
C31	0.0219 (9)	0.0241 (10)	0.0332 (11)	−0.0022 (8)	−0.0071 (8)	−0.0027 (8)
C32	0.0431 (13)	0.0203 (10)	0.0345 (11)	−0.0020 (9)	−0.0035 (9)	−0.0068 (8)
C33	0.0317 (11)	0.0337 (11)	0.0385 (12)	−0.0148 (9)	−0.0111 (9)	0.0056 (9)
C34	0.0277 (10)	0.0249 (10)	0.0251 (10)	0.0040 (8)	−0.0026 (8)	−0.0031 (8)
C35	0.0299 (11)	0.0288 (10)	0.0247 (10)	0.0005 (8)	−0.0057 (8)	0.0007 (8)
C36	0.0343 (11)	0.0281 (10)	0.0221 (9)	−0.0087 (8)	−0.0045 (8)	−0.0054 (8)
N41	0.0239 (8)	0.0223 (8)	0.0236 (8)	−0.0054 (6)	−0.0049 (6)	0.0000 (6)
N42	0.0301 (10)	0.0273 (9)	0.0417 (11)	0.0018 (7)	0.0013 (8)	−0.0037 (8)
N43	0.0397 (11)	0.0287 (10)	0.0452 (11)	−0.0112 (8)	−0.0241 (9)	0.0100 (8)
N44	0.0273 (9)	0.0219 (8)	0.0310 (9)	−0.0063 (7)	−0.0025 (7)	0.0009 (7)
C41	0.0304 (11)	0.0307 (11)	0.0281 (10)	−0.0011 (9)	0.0019 (8)	−0.0010 (9)
C42	0.0240 (11)	0.0309 (12)	0.0623 (17)	−0.0016 (9)	−0.0088 (10)	0.0084 (11)
C43	0.0447 (13)	0.0295 (11)	0.0302 (11)	−0.0087 (10)	−0.0126 (10)	0.0075 (9)
C44	0.0246 (10)	0.0231 (9)	0.0276 (10)	−0.0028 (8)	−0.0034 (8)	−0.0004 (8)
C45	0.0420 (13)	0.0243 (10)	0.0404 (12)	−0.0122 (9)	−0.0219 (10)	0.0056 (9)
C46	0.0343 (11)	0.0199 (10)	0.0434 (13)	−0.0050 (8)	−0.0042 (10)	−0.0059 (9)

### Bis(ethanol-κO)bis(hexamethylenetetramine-κN)bis(thiocyanato-κN)cobalt(II)–diaqua-κ2O-bis(hexamethylenetetramine-κN)bis(thiocyanato-κN)cobalt(II)–ethanol–hexamethylenetetramine (1.2/0.8/1.6/4) (1) Geometric parameters (Å, º)

**Table:**

Co1—N1^i^	2.0590 (16)	C16—H16B	0.9700
Co1—N1	2.0590 (16)	N21—C21	1.493 (2)
Co1—O1^i^	2.1388 (13)	N21—C24	1.490 (2)
Co1—O1	2.1388 (13)	N21—C25	1.500 (2)
Co1—N11^i^	2.2834 (15)	N22—C23	1.478 (2)
Co1—N11	2.2834 (15)	N22—C24	1.474 (2)
Co2—N2^ii^	2.0812 (16)	N22—C26	1.469 (2)
Co2—N2	2.0812 (16)	N23—C21	1.465 (2)
Co2—O2	2.029 (6)	N23—C22	1.468 (2)
Co2—O2^ii^	2.029 (6)	N23—C26	1.469 (2)
Co2—O4^ii^	2.21 (3)	N24—C22	1.473 (2)
Co2—O4	2.21 (3)	N24—C23	1.470 (2)
Co2—N21	2.2788 (16)	N24—C25	1.465 (2)
Co2—N21^ii^	2.2788 (16)	C21—H21A	0.9700
N1—C1	1.169 (3)	C21—H21B	0.9700
C1—S1	1.629 (2)	C22—H22A	0.9700
N2—C2	1.154 (3)	C22—H22B	0.9700
C2—S2	1.643 (2)	C23—H23A	0.9700
O1—H1	0.880 (18)	C23—H23B	0.9700
O1—C3	1.464 (2)	C24—H24A	0.9700
C3—H3AA	0.9700	C24—H24B	0.9700
C3—H3AB	0.9700	C25—H25A	0.9700
C3—H3BC	0.9700	C25—H25B	0.9700
C3—H3BD	0.9700	C26—H26A	0.9700
C3—C4	1.515 (3)	C26—H26B	0.9700
C3—C4'	1.4495 (10)	N31—C31	1.486 (2)
C4—H4A	0.9600	N31—C34	1.474 (3)
C4—H4B	0.9600	N31—C36	1.486 (2)
C4—H4C	0.9600	N32—C31	1.466 (3)
C4'—H4'A	0.9600	N32—C32	1.471 (3)
C4'—H4'B	0.9600	N32—C35	1.465 (3)
C4'—H4'C	0.9600	N33—C32	1.469 (3)
O2—H2A	0.872 (19)	N33—C33	1.467 (3)
O2—H2B	0.871 (19)	N33—C36	1.463 (3)
O3—C5	1.433 (3)	N34—C33	1.480 (3)
O3—H3	0.871 (19)	N34—C34	1.470 (3)
C5—H5A	0.9700	N34—C35	1.480 (3)
C5—H5B	0.9700	C31—H31A	0.9700
C5—C6	1.505 (4)	C31—H31B	0.9700
C6—H6A	0.9600	C32—H32A	0.9700
C6—H6B	0.9600	C32—H32B	0.9700
C6—H6C	0.9600	C33—H33A	0.9700
O4—C7	1.427 (17)	C33—H33B	0.9700
O4—H4	0.87 (2)	C34—H34A	0.9700
C7—H7A	0.9700	C34—H34B	0.9700
C7—H7B	0.9700	C35—H35A	0.9700
C7—C8	1.507 (13)	C35—H35B	0.9700
C8—H8A	0.9600	C36—H36A	0.9700
C8—H8B	0.9600	C36—H36B	0.9700
C8—H8C	0.9600	N41—C41	1.483 (3)
N11—C11	1.499 (2)	N41—C44	1.482 (2)
N11—C12	1.488 (2)	N41—C45	1.475 (3)
N11—C15	1.496 (2)	N42—C41	1.465 (3)
N12—C11	1.465 (2)	N42—C42	1.469 (3)
N12—C14	1.472 (2)	N42—C46	1.464 (3)
N12—C16	1.476 (2)	N43—C42	1.479 (4)
N13—C13	1.474 (2)	N43—C43	1.473 (3)
N13—C14	1.470 (2)	N43—C45	1.469 (3)
N13—C15	1.468 (2)	N44—C43	1.465 (3)
N14—C12	1.467 (2)	N44—C44	1.467 (3)
N14—C13	1.472 (2)	N44—C46	1.463 (3)
N14—C16	1.473 (2)	C41—H41A	0.9700
C11—H11A	0.9700	C41—H41B	0.9700
C11—H11B	0.9700	C42—H42A	0.9700
C12—H12A	0.9700	C42—H42B	0.9700
C12—H12B	0.9700	C43—H43A	0.9700
C13—H13A	0.9700	C43—H43B	0.9700
C13—H13B	0.9700	C44—H44A	0.9700
C14—H14A	0.9700	C44—H44B	0.9700
C14—H14B	0.9700	C45—H45A	0.9700
C15—H15A	0.9700	C45—H45B	0.9700
C15—H15B	0.9700	C46—H46A	0.9700
C16—H16A	0.9700	C46—H46B	0.9700
			
N1^i^—Co1—N1	180.0	N14—C16—H16A	109.1
N1—Co1—O1^i^	89.16 (6)	N14—C16—H16B	109.1
N1^i^—Co1—O1	89.16 (6)	H16A—C16—H16B	107.9
N1—Co1—O1	90.84 (6)	C21—N21—Co2	110.79 (11)
N1^i^—Co1—O1^i^	90.84 (6)	C21—N21—C25	106.97 (14)
N1^i^—Co1—N11	93.90 (6)	C24—N21—Co2	113.66 (11)
N1—Co1—N11	86.10 (6)	C24—N21—C21	107.30 (14)
N1—Co1—N11^i^	93.90 (6)	C24—N21—C25	107.16 (14)
N1^i^—Co1—N11^i^	86.10 (6)	C25—N21—Co2	110.66 (11)
O1—Co1—O1^i^	180.00 (7)	C24—N22—C23	108.01 (14)
O1^i^—Co1—N11^i^	91.57 (5)	C26—N22—C23	107.79 (15)
O1—Co1—N11	91.57 (5)	C26—N22—C24	108.16 (15)
O1—Co1—N11^i^	88.43 (5)	C21—N23—C22	108.62 (14)
O1^i^—Co1—N11	88.43 (5)	C21—N23—C26	108.13 (15)
N11—Co1—N11^i^	180.0	C22—N23—C26	107.86 (15)
N2—Co2—N2^ii^	180.0	C23—N24—C22	108.30 (14)
N2—Co2—O4	85.7 (7)	C25—N24—C22	107.77 (15)
N2^ii^—Co2—O4^ii^	85.7 (7)	C25—N24—C23	107.98 (15)
N2—Co2—O4^ii^	94.3 (7)	N21—C21—H21A	109.1
N2^ii^—Co2—O4	94.3 (7)	N21—C21—H21B	109.1
N2—Co2—N21^ii^	91.78 (6)	N23—C21—N21	112.54 (15)
N2^ii^—Co2—N21^ii^	88.22 (6)	N23—C21—H21A	109.1
N2^ii^—Co2—N21	91.78 (6)	N23—C21—H21B	109.1
N2—Co2—N21	88.22 (6)	H21A—C21—H21B	107.8
O2^ii^—Co2—N2	89.20 (16)	N23—C22—N24	112.53 (15)
O2—Co2—N2^ii^	89.20 (16)	N23—C22—H22A	109.1
O2^ii^—Co2—N2^ii^	90.80 (16)	N23—C22—H22B	109.1
O2—Co2—N2	90.80 (16)	N24—C22—H22A	109.1
O2^ii^—Co2—O2	180.0	N24—C22—H22B	109.1
O2—Co2—O4^ii^	174.6 (9)	H22A—C22—H22B	107.8
O2^ii^—Co2—O4^ii^	5.4 (9)	N22—C23—H23A	109.1
O2—Co2—N21	91.3 (2)	N22—C23—H23B	109.1
O2—Co2—N21^ii^	88.7 (2)	N24—C23—N22	112.47 (15)
O2^ii^—Co2—N21^ii^	91.3 (2)	N24—C23—H23A	109.1
O2^ii^—Co2—N21	88.7 (2)	N24—C23—H23B	109.1
O4^ii^—Co2—O4	180.0	H23A—C23—H23B	107.8
O4^ii^—Co2—N21^ii^	92.9 (9)	N21—C24—H24A	109.1
O4—Co2—N21	92.9 (9)	N21—C24—H24B	109.1
O4^ii^—Co2—N21	87.1 (9)	N22—C24—N21	112.52 (15)
O4—Co2—N21^ii^	87.1 (9)	N22—C24—H24A	109.1
N21^ii^—Co2—N21	180.0	N22—C24—H24B	109.1
C1—N1—Co1	164.85 (15)	H24A—C24—H24B	107.8
N1—C1—S1	178.89 (18)	N21—C25—H25A	109.0
C2—N2—Co2	175.29 (16)	N21—C25—H25B	109.0
N2—C2—S2	177.31 (19)	N24—C25—N21	112.98 (14)
Co1—O1—H1	122 (2)	N24—C25—H25A	109.0
C3—O1—Co1	129.84 (12)	N24—C25—H25B	109.0
C3—O1—H1	107 (2)	H25A—C25—H25B	107.8
O1—C3—H3AA	109.0	N22—C26—H26A	109.0
O1—C3—H3AB	109.0	N22—C26—H26B	109.0
O1—C3—H3BC	106.0	N23—C26—N22	112.80 (15)
O1—C3—H3BD	106.0	N23—C26—H26A	109.0
O1—C3—C4	113.01 (18)	N23—C26—H26B	109.0
H3AA—C3—H3AB	107.8	H26A—C26—H26B	107.8
H3BC—C3—H3BD	106.3	C34—N31—C31	107.28 (15)
C4—C3—H3AA	109.0	C34—N31—C36	107.97 (16)
C4—C3—H3AB	109.0	C36—N31—C31	107.86 (16)
C4'—C3—O1	125.1 (6)	C31—N32—C32	107.40 (17)
C4'—C3—H3BC	106.0	C35—N32—C31	107.85 (16)
C4'—C3—H3BD	106.0	C35—N32—C32	108.44 (17)
C3—C4—H4A	109.5	C33—N33—C32	108.52 (17)
C3—C4—H4B	109.5	C36—N33—C32	108.32 (17)
C3—C4—H4C	109.5	C36—N33—C33	108.01 (18)
H4A—C4—H4B	109.5	C34—N34—C33	107.50 (16)
H4A—C4—H4C	109.5	C34—N34—C35	108.18 (16)
H4B—C4—H4C	109.5	C35—N34—C33	107.51 (17)
C3—C4'—H4'A	109.5	N31—C31—H31A	109.0
C3—C4'—H4'B	109.5	N31—C31—H31B	109.0
C3—C4'—H4'C	109.5	N32—C31—N31	112.86 (16)
H4'A—C4'—H4'B	109.5	N32—C31—H31A	109.0
H4'A—C4'—H4'C	109.5	N32—C31—H31B	109.0
H4'B—C4'—H4'C	109.5	H31A—C31—H31B	107.8
Co2—O2—H2A	127 (4)	N32—C32—H32A	109.1
Co2—O2—H2B	123 (3)	N32—C32—H32B	109.1
H2A—O2—H2B	106 (4)	N33—C32—N32	112.59 (17)
C5—O3—H3	112 (3)	N33—C32—H32A	109.1
O3—C5—H5A	109.8	N33—C32—H32B	109.1
O3—C5—H5B	109.8	H32A—C32—H32B	107.8
O3—C5—C6	109.6 (2)	N33—C33—N34	112.55 (17)
H5A—C5—H5B	108.2	N33—C33—H33A	109.1
C6—C5—H5A	109.8	N33—C33—H33B	109.1
C6—C5—H5B	109.8	N34—C33—H33A	109.1
C5—C6—H6A	109.5	N34—C33—H33B	109.1
C5—C6—H6B	109.5	H33A—C33—H33B	107.8
C5—C6—H6C	109.5	N31—C34—H34A	109.1
H6A—C6—H6B	109.5	N31—C34—H34B	109.1
H6A—C6—H6C	109.5	N34—C34—N31	112.62 (16)
H6B—C6—H6C	109.5	N34—C34—H34A	109.1
Co2—O4—H4	115 (10)	N34—C34—H34B	109.1
C7—O4—Co2	137 (2)	H34A—C34—H34B	107.8
C7—O4—H4	106 (10)	N32—C35—N34	112.61 (16)
O4—C7—H7A	109.1	N32—C35—H35A	109.1
O4—C7—H7B	109.1	N32—C35—H35B	109.1
O4—C7—C8	112.4 (15)	N34—C35—H35A	109.1
H7A—C7—H7B	107.9	N34—C35—H35B	109.1
C8—C7—H7A	109.1	H35A—C35—H35B	107.8
C8—C7—H7B	109.1	N31—C36—H36A	109.2
C7—C8—H8A	109.5	N31—C36—H36B	109.2
C7—C8—H8B	109.5	N33—C36—N31	111.87 (16)
C7—C8—H8C	109.5	N33—C36—H36A	109.2
H8A—C8—H8B	109.5	N33—C36—H36B	109.2
H8A—C8—H8C	109.5	H36A—C36—H36B	107.9
H8B—C8—H8C	109.5	C44—N41—C41	107.46 (16)
C11—N11—Co1	112.38 (10)	C45—N41—C41	108.17 (18)
C12—N11—Co1	110.45 (11)	C45—N41—C44	107.65 (16)
C12—N11—C11	106.96 (14)	C41—N42—C42	107.76 (19)
C12—N11—C15	106.95 (13)	C46—N42—C41	107.89 (17)
C15—N11—Co1	112.93 (11)	C46—N42—C42	107.80 (19)
C15—N11—C11	106.83 (14)	C43—N43—C42	107.73 (19)
C11—N12—C14	108.22 (14)	C45—N43—C42	108.42 (19)
C11—N12—C16	108.09 (14)	C45—N43—C43	107.79 (19)
C14—N12—C16	108.03 (14)	C43—N44—C44	108.03 (17)
C14—N13—C13	107.97 (15)	C46—N44—C43	108.19 (18)
C15—N13—C13	107.82 (14)	C46—N44—C44	107.81 (16)
C15—N13—C14	108.50 (14)	N41—C41—H41A	109.1
C12—N14—C13	108.45 (14)	N41—C41—H41B	109.1
C12—N14—C16	108.30 (14)	N42—C41—N41	112.64 (17)
C13—N14—C16	107.44 (14)	N42—C41—H41A	109.1
N11—C11—H11A	109.0	N42—C41—H41B	109.1
N11—C11—H11B	109.0	H41A—C41—H41B	107.8
N12—C11—N11	113.01 (14)	N42—C42—N43	112.48 (18)
N12—C11—H11A	109.0	N42—C42—H42A	109.1
N12—C11—H11B	109.0	N42—C42—H42B	109.1
H11A—C11—H11B	107.8	N43—C42—H42A	109.1
N11—C12—H12A	109.0	N43—C42—H42B	109.1
N11—C12—H12B	109.0	H42A—C42—H42B	107.8
N14—C12—N11	112.97 (15)	N43—C43—H43A	109.1
N14—C12—H12A	109.0	N43—C43—H43B	109.1
N14—C12—H12B	109.0	N44—C43—N43	112.46 (18)
H12A—C12—H12B	107.8	N44—C43—H43A	109.1
N13—C13—H13A	109.1	N44—C43—H43B	109.1
N13—C13—H13B	109.1	H43A—C43—H43B	107.8
N14—C13—N13	112.61 (14)	N41—C44—H44A	109.1
N14—C13—H13A	109.1	N41—C44—H44B	109.1
N14—C13—H13B	109.1	N44—C44—N41	112.30 (16)
H13A—C13—H13B	107.8	N44—C44—H44A	109.1
N12—C14—H14A	109.1	N44—C44—H44B	109.1
N12—C14—H14B	109.1	H44A—C44—H44B	107.9
N13—C14—N12	112.35 (15)	N41—C45—H45A	109.2
N13—C14—H14A	109.1	N41—C45—H45B	109.2
N13—C14—H14B	109.1	N43—C45—N41	112.17 (17)
H14A—C14—H14B	107.9	N43—C45—H45A	109.2
N11—C15—H15A	109.0	N43—C45—H45B	109.2
N11—C15—H15B	109.0	H45A—C45—H45B	107.9
N13—C15—N11	112.95 (14)	N42—C46—H46A	108.9
N13—C15—H15A	109.0	N42—C46—H46B	108.9
N13—C15—H15B	109.0	N44—C46—N42	113.21 (17)
H15A—C15—H15B	107.8	N44—C46—H46A	108.9
N12—C16—H16A	109.1	N44—C46—H46B	108.9
N12—C16—H16B	109.1	H46A—C46—H46B	107.7
N14—C16—N12	112.42 (15)		
			
Co1—O1—C3—C4	121.56 (18)	C26—N22—C23—N24	−57.6 (2)
Co1—O1—C3—C4'	85.5 (9)	C26—N22—C24—N21	57.87 (19)
Co1—N11—C11—N12	−178.62 (11)	C26—N23—C21—N21	−58.52 (19)
Co1—N11—C12—N14	179.71 (11)	C26—N23—C22—N24	58.1 (2)
Co1—N11—C15—N13	179.36 (12)	C31—N31—C34—N34	57.9 (2)
Co2—O4—C7—C8	125 (2)	C31—N31—C36—N33	−57.3 (2)
Co2—N21—C21—N23	−177.58 (11)	C31—N32—C32—N33	58.9 (2)
Co2—N21—C24—N22	179.79 (11)	C31—N32—C35—N34	−58.0 (2)
Co2—N21—C25—N24	178.24 (12)	C32—N32—C31—N31	−58.1 (2)
C11—N11—C12—N14	57.12 (18)	C32—N32—C35—N34	58.0 (2)
C11—N11—C15—N13	−56.60 (19)	C32—N33—C33—N34	−57.9 (2)
C11—N12—C14—N13	58.77 (19)	C32—N33—C36—N31	58.5 (2)
C11—N12—C16—N14	−58.43 (18)	C33—N33—C32—N32	57.4 (2)
C12—N11—C11—N12	−57.24 (19)	C33—N33—C36—N31	−58.9 (2)
C12—N11—C15—N13	57.65 (19)	C33—N34—C34—N31	57.9 (2)
C12—N14—C13—N13	−58.23 (19)	C33—N34—C35—N32	−58.0 (2)
C12—N14—C16—N12	58.46 (19)	C34—N31—C31—N32	−58.4 (2)
C13—N13—C14—N12	58.01 (19)	C34—N31—C36—N33	58.3 (2)
C13—N13—C15—N11	−58.61 (19)	C34—N34—C33—N33	−58.4 (2)
C13—N14—C12—N11	57.91 (19)	C34—N34—C35—N32	57.8 (2)
C13—N14—C16—N12	−58.50 (19)	C35—N32—C31—N31	58.6 (2)
C14—N12—C11—N11	−58.54 (19)	C35—N32—C32—N33	−57.4 (2)
C14—N12—C16—N14	58.45 (19)	C35—N34—C33—N33	57.9 (2)
C14—N13—C13—N14	−58.58 (19)	C35—N34—C34—N31	−58.0 (2)
C14—N13—C15—N11	58.09 (19)	C36—N31—C31—N32	57.7 (2)
C15—N11—C11—N12	57.00 (19)	C36—N31—C34—N34	−58.1 (2)
C15—N11—C12—N14	−57.03 (18)	C36—N33—C32—N32	−59.6 (2)
C15—N13—C13—N14	58.46 (19)	C36—N33—C33—N34	59.3 (2)
C15—N13—C14—N12	−58.6 (2)	C41—N41—C44—N44	58.1 (2)
C16—N12—C11—N11	58.22 (19)	C41—N41—C45—N43	−57.3 (2)
C16—N12—C14—N13	−58.04 (19)	C41—N42—C42—N43	58.4 (2)
C16—N14—C12—N11	−58.39 (19)	C41—N42—C46—N44	−58.3 (2)
C16—N14—C13—N13	58.63 (19)	C42—N42—C41—N41	−58.4 (2)
C21—N21—C24—N22	−57.37 (19)	C42—N42—C46—N44	57.9 (2)
C21—N21—C25—N24	57.47 (19)	C42—N43—C43—N44	−58.0 (2)
C21—N23—C22—N24	−58.9 (2)	C42—N43—C45—N41	57.5 (2)
C21—N23—C26—N22	58.7 (2)	C43—N43—C42—N42	58.1 (2)
C22—N23—C21—N21	58.28 (19)	C43—N43—C45—N41	−58.8 (3)
C22—N23—C26—N22	−58.6 (2)	C43—N44—C44—N41	58.3 (2)
C22—N24—C23—N22	57.5 (2)	C43—N44—C46—N42	−57.9 (2)
C22—N24—C25—N21	−58.47 (19)	C44—N41—C41—N42	−57.8 (2)
C23—N22—C24—N21	−58.54 (19)	C44—N41—C45—N43	58.5 (2)
C23—N22—C26—N23	58.2 (2)	C44—N44—C43—N43	−58.6 (2)
C23—N24—C22—N23	−57.9 (2)	C44—N44—C46—N42	58.7 (2)
C23—N24—C25—N21	58.31 (19)	C45—N41—C41—N42	58.1 (2)
C24—N21—C21—N23	57.83 (19)	C45—N41—C44—N44	−58.2 (2)
C24—N21—C25—N24	−57.34 (19)	C45—N43—C42—N42	−58.3 (2)
C24—N22—C23—N24	59.1 (2)	C45—N43—C43—N44	58.8 (3)
C24—N22—C26—N23	−58.31 (19)	C46—N42—C41—N41	57.7 (2)
C25—N21—C21—N23	−56.89 (18)	C46—N42—C42—N43	−57.8 (2)
C25—N21—C24—N22	57.21 (19)	C46—N44—C43—N43	57.8 (2)
C25—N24—C22—N23	58.72 (19)	C46—N44—C44—N41	−58.4 (2)
C25—N24—C23—N22	−58.9 (2)		

Symmetry codes: (i) −*x*+1, −*y*, −*z*+2; (ii) −*x*+2, −*y*+1, −*z*+1.

### Bis(ethanol-κO)bis(hexamethylenetetramine-κN)bis(thiocyanato-κN)cobalt(II)–diaqua-κ2O-bis(hexamethylenetetramine-κN)bis(thiocyanato-κN)cobalt(II)–ethanol–hexamethylenetetramine (1.2/0.8/1.6/4) (1) Hydrogen-bond geometry (Å, º)

**Table:**

*D*—H···*A*	*D*—H	H···*A*	*D*···*A*	*D*—H···*A*
O1—H1···N31	0.88 (2)	1.92 (2)	2.793 (2)	170 (3)
C4′—H4′*A*···N43^iii^	0.96	2.50	3.243 (14)	134
C4′—H4′*C*···N44^i^	0.96	2.38	3.161 (10)	138
O2—H2*A*···N41	0.87 (2)	1.88 (2)	2.743 (7)	173 (7)
O2—H2*B*···O3	0.87 (2)	1.80 (2)	2.665 (4)	177 (5)
C5—H5*A*···S2^iv^	0.97	3.02	3.925 (3)	156
O3—H3···N34	0.87 (2)	1.97 (2)	2.821 (3)	167 (4)
O4—H4···N41	0.87 (2)	1.94 (5)	2.81 (3)	170 (19)
C11—H11*A*···O1^i^	0.97	2.49	3.058 (2)	117
C11—H11*B*···N1	0.97	2.67	3.213 (2)	116
C12—H12*B*···N44	0.97	2.64	3.423 (3)	138
C13—H13*A*···N13^v^	0.97	2.70	3.563 (2)	149
C13—H13*B*···S2^iv^	0.97	2.95	3.7150 (19)	136
C14—H14*A*···S2^vi^	0.97	2.93	3.840 (2)	156
C15—H15*B*···O1	0.97	2.61	3.118 (2)	113
C22—H22*B*···N12^vii^	0.97	2.58	3.448 (2)	149
C25—H25*A*···O2^ii^	0.97	2.50	3.026 (7)	114
C25—H25*A*···O4^ii^	0.97	2.49	3.08 (3)	119
C25—H25*B*···N2	0.97	2.61	3.202 (3)	119
C26—H26*A*···S1^i^	0.97	2.98	3.655 (2)	128
C26—H26*B*···N22^viii^	0.97	2.69	3.581 (3)	152
C33—H33*A*···N23	0.97	2.66	3.431 (3)	137
C45—H45*A*···S2^ii^	0.97	3.01	3.959 (3)	165

Symmetry codes: (i) −*x*+1, −*y*, −*z*+2; (ii) −*x*+2, −*y*+1, −*z*+1; (iii) *x*−1, *y*, *z*; (iv) −*x*+1, −*y*+1, −*z*+1; (v) −*x*+1, −*y*, −*z*+1; (vi) *x*, *y*−1, *z*; (vii) *x*, *y*+1, *z*; (viii) −*x*+2, −*y*+1, −*z*+2.

### \ Tris(ethanol-κO)(hexamethylenetetramine-κN)bis(thiocyanato-\ κN)cobalt(II) (2) Crystal data

**Table:**

[Co(NCS)_2_(C_6_H_12_N_4_)(C_2_H_6_O)_3_]	*F*(000) = 956
*M**_r_* = 453.49	*D*_x_ = 1.449 Mg m^−^^3^
Monoclinic, *P*2_1_/*n*	Cu *K*α radiation, λ = 1.54184 Å
*a* = 11.1463 (1) Å	Cell parameters from 23697 reflections
*b* = 15.7705 (1) Å	θ = 4.7–78.0°
*c* = 12.1824 (1) Å	µ = 8.57 mm^−^^1^
β = 103.886 (1)°	*T* = 100 K
*V* = 2078.87 (3) Å^3^	Block, intense orange
*Z* = 4	0.2 × 0.18 × 0.03 mm

### \ Tris(ethanol-κO)(hexamethylenetetramine-κN)bis(thiocyanato-\ κN)cobalt(II) (2) Data collection

**Table:**

XtaLAB Synergy, Dualflex, HyPix diffractometer	4431 independent reflections
Radiation source: micro-focus sealed X-ray tube, PhotonJet (Cu) X-ray Source	4373 reflections with *I* > 2σ(*I*)
Mirror monochromator	*R*_int_ = 0.027
Detector resolution: 10.0000 pixels mm^-1^	θ_max_ = 78.2°, θ_min_ = 4.7°
ω scans	*h* = −14→13
Absorption correction: multi-scan (CrysAlisPro; Rigaku OD, 2021)	*k* = −20→18
*T*_min_ = 0.427, *T*_max_ = 1.000	*l* = −14→15
29441 measured reflections	

### \ Tris(ethanol-κO)(hexamethylenetetramine-κN)bis(thiocyanato-\ κN)cobalt(II) (2) Refinement

**Table:**

Refinement on *F*^2^	Hydrogen site location: inferred from neighbouring sites
Least-squares matrix: full	H-atom parameters constrained
*R*[*F*^2^ > 2σ(*F*^2^)] = 0.025	*w* = 1/[σ^2^(*F*_o_^2^) + (0.0403*P*)^2^ + 0.8765*P*] where *P* = (*F*_o_^2^ + 2*F*_c_^2^)/3
*wR*(*F*^2^) = 0.068	(Δ/σ)_max_ = 0.002
*S* = 1.08	Δρ_max_ = 0.32 e Å^−^^3^
4431 reflections	Δρ_min_ = −0.31 e Å^−^^3^
242 parameters	Extinction correction: SHELXL2016/6 (Sheldrick 2015b), Fc^*^=kFc[1+0.001xFc^2^λ^3^/sin(2θ)]^-1/4^
1 restraint	Extinction coefficient: 0.00065 (9)
Primary atom site location: dual	

### \ Tris(ethanol-κO)(hexamethylenetetramine-κN)bis(thiocyanato-\ κN)cobalt(II) (2) Special details

**Table:**

Geometry. All esds (except the esd in the dihedral angle between two l.s. planes) are estimated using the full covariance matrix. The cell esds are taken into account individually in the estimation of esds in distances, angles and torsion angles; correlations between esds in cell parameters are only used when they are defined by crystal symmetry. An approximate (isotropic) treatment of cell esds is used for estimating esds involving l.s. planes.

### \ Tris(ethanol-κO)(hexamethylenetetramine-κN)bis(thiocyanato-\ κN)cobalt(II) (2) Fractional atomic coordinates and isotropic or equivalent isotropic displacement parameters (Å^2^)

**Table:**

	*x*	*y*	*z*	*U*_iso_*/*U*_eq_	
Co1	0.51335 (2)	0.27266 (2)	0.57458 (2)	0.01059 (7)	
N1	0.67247 (10)	0.34371 (7)	0.62465 (9)	0.0153 (2)	
C1	0.76466 (12)	0.38035 (8)	0.65618 (11)	0.0141 (2)	
S1	0.89474 (3)	0.43141 (2)	0.70092 (3)	0.02407 (10)	
N2	0.35462 (10)	0.20093 (7)	0.52823 (9)	0.0153 (2)	
C2	0.25674 (12)	0.17255 (8)	0.49267 (11)	0.0134 (2)	
S2	0.11817 (3)	0.13335 (2)	0.44031 (3)	0.01552 (8)	
N11	0.48544 (9)	0.32562 (7)	0.39886 (9)	0.0110 (2)	
N12	0.35142 (10)	0.34740 (7)	0.20867 (9)	0.0131 (2)	
N13	0.49385 (10)	0.45980 (7)	0.29661 (9)	0.0127 (2)	
N14	0.57416 (10)	0.32908 (7)	0.23263 (9)	0.0137 (2)	
C11	0.36212 (11)	0.30856 (8)	0.32095 (10)	0.0123 (2)	
H11A	0.349734	0.246546	0.312045	0.015*	
H11B	0.296401	0.331440	0.354686	0.015*	
C12	0.37105 (12)	0.44001 (8)	0.22303 (11)	0.0140 (2)	
H12A	0.363639	0.466531	0.148044	0.017*	
H12B	0.306101	0.464431	0.256487	0.017*	
C13	0.58915 (12)	0.42110 (8)	0.24547 (11)	0.0147 (2)	
H13A	0.583835	0.446900	0.170375	0.018*	
H13B	0.672136	0.433681	0.293783	0.018*	
C14	0.58124 (11)	0.29098 (8)	0.34359 (11)	0.0130 (2)	
H14A	0.664260	0.301634	0.393063	0.016*	
H14B	0.570316	0.228850	0.334757	0.016*	
C15	0.45097 (12)	0.31188 (8)	0.15994 (11)	0.0147 (2)	
H15A	0.439358	0.249853	0.150305	0.018*	
H15B	0.445047	0.337046	0.084374	0.018*	
C16	0.50169 (12)	0.41931 (8)	0.40684 (11)	0.0126 (2)	
H16A	0.437357	0.443759	0.441062	0.015*	
H16B	0.583193	0.432292	0.457605	0.015*	
O21	0.41718 (8)	0.37678 (6)	0.62519 (8)	0.01481 (19)	
H21	0.458750	0.419051	0.654234	0.022*	
C21	0.31119 (12)	0.36740 (9)	0.67299 (12)	0.0174 (3)	
H21A	0.320943	0.404600	0.740012	0.021*	
H21B	0.306126	0.308048	0.697966	0.021*	
C22	0.19389 (14)	0.39014 (12)	0.58774 (14)	0.0295 (3)	
H22A	0.181805	0.351233	0.523285	0.044*	
H22B	0.199742	0.448405	0.561561	0.044*	
H22C	0.123733	0.385578	0.622869	0.044*	
O31	0.62924 (8)	0.17312 (6)	0.54561 (8)	0.01390 (18)	
H31	0.695985	0.172350	0.595461	0.021*	
C31	0.59743 (13)	0.08809 (8)	0.50586 (12)	0.0172 (3)	
H31A	0.508810	0.085995	0.466374	0.021*	
H31B	0.610588	0.049076	0.571334	0.021*	
C32	0.67444 (14)	0.05882 (9)	0.42600 (12)	0.0210 (3)	
H32A	0.658634	0.095774	0.359399	0.032*	
H32B	0.652206	0.000353	0.402320	0.032*	
H32C	0.762264	0.061398	0.464640	0.032*	
O41	0.54084 (10)	0.22913 (6)	0.74187 (8)	0.0173 (2)	
H41	0.564436	0.261997	0.797094	0.026*	
C41	0.51429 (13)	0.14737 (9)	0.78184 (12)	0.0197 (3)	
H41A	0.591114	0.123102	0.829704	0.024*	
H41B	0.485447	0.109128	0.716372	0.024*	
C42	0.41750 (15)	0.15144 (12)	0.84905 (16)	0.0344 (4)	
H42A	0.339647	0.171959	0.800591	0.052*	
H42B	0.444763	0.190262	0.912993	0.052*	
H42C	0.405042	0.094752	0.877347	0.052*	

### \ Tris(ethanol-κO)(hexamethylenetetramine-κN)bis(thiocyanato-\ κN)cobalt(II) (2) Atomic displacement parameters (Å^2^)

**Table:**

	*U*^11^	*U*^22^	*U*^33^	*U*^12^	*U*^13^	*U*^23^
Co1	0.00865 (11)	0.01120 (12)	0.01104 (12)	−0.00058 (7)	0.00060 (8)	−0.00032 (7)
N1	0.0121 (5)	0.0164 (5)	0.0155 (5)	−0.0011 (4)	−0.0003 (4)	−0.0007 (4)
C1	0.0149 (6)	0.0116 (6)	0.0154 (6)	0.0034 (5)	0.0029 (5)	0.0009 (5)
S1	0.01192 (16)	0.01850 (17)	0.0400 (2)	−0.00438 (12)	0.00274 (14)	−0.00662 (14)
N2	0.0143 (5)	0.0149 (5)	0.0157 (5)	−0.0015 (4)	0.0016 (4)	0.0008 (4)
C2	0.0157 (6)	0.0123 (6)	0.0122 (5)	0.0019 (5)	0.0035 (5)	0.0015 (4)
S2	0.01174 (15)	0.01751 (16)	0.01595 (15)	−0.00302 (11)	0.00064 (11)	−0.00024 (11)
N11	0.0099 (5)	0.0110 (5)	0.0114 (5)	−0.0007 (4)	0.0014 (4)	−0.0008 (4)
N12	0.0133 (5)	0.0133 (5)	0.0117 (5)	−0.0004 (4)	0.0015 (4)	0.0004 (4)
N13	0.0117 (5)	0.0125 (5)	0.0132 (5)	−0.0006 (4)	0.0018 (4)	0.0005 (4)
N14	0.0135 (5)	0.0143 (5)	0.0137 (5)	−0.0003 (4)	0.0038 (4)	0.0003 (4)
C11	0.0101 (5)	0.0146 (6)	0.0111 (6)	−0.0015 (4)	0.0006 (4)	0.0010 (4)
C12	0.0117 (6)	0.0128 (6)	0.0156 (6)	0.0005 (4)	−0.0003 (5)	0.0017 (5)
C13	0.0126 (6)	0.0150 (6)	0.0169 (6)	−0.0014 (5)	0.0044 (5)	0.0002 (5)
C14	0.0116 (6)	0.0145 (6)	0.0134 (6)	0.0029 (5)	0.0037 (5)	0.0006 (5)
C15	0.0157 (6)	0.0157 (6)	0.0127 (6)	−0.0009 (5)	0.0037 (5)	−0.0018 (5)
C16	0.0131 (6)	0.0115 (6)	0.0124 (5)	−0.0006 (4)	0.0017 (5)	−0.0010 (4)
O21	0.0123 (4)	0.0142 (4)	0.0191 (5)	−0.0019 (3)	0.0062 (4)	−0.0027 (3)
C21	0.0156 (6)	0.0202 (7)	0.0183 (6)	−0.0013 (5)	0.0078 (5)	−0.0025 (5)
C22	0.0155 (7)	0.0410 (9)	0.0313 (8)	0.0045 (6)	0.0044 (6)	−0.0042 (7)
O31	0.0114 (4)	0.0129 (4)	0.0148 (4)	0.0013 (3)	−0.0020 (3)	−0.0017 (3)
C31	0.0169 (6)	0.0139 (6)	0.0190 (6)	−0.0001 (5)	0.0007 (5)	−0.0031 (5)
C32	0.0215 (7)	0.0214 (7)	0.0175 (6)	0.0063 (5)	−0.0008 (5)	−0.0041 (5)
O41	0.0230 (5)	0.0156 (5)	0.0122 (4)	−0.0048 (3)	0.0022 (4)	−0.0001 (3)
C41	0.0209 (7)	0.0159 (6)	0.0192 (6)	−0.0026 (5)	−0.0011 (5)	0.0040 (5)
C42	0.0219 (8)	0.0392 (9)	0.0434 (10)	−0.0028 (7)	0.0103 (7)	0.0176 (8)

### \ Tris(ethanol-κO)(hexamethylenetetramine-κN)bis(thiocyanato-\ κN)cobalt(II) (2) Geometric parameters (Å, º)

**Table:**

Co1—N2	2.0615 (11)	C14—H14B	0.9900
Co1—N1	2.0624 (11)	C15—H15A	0.9900
Co1—O41	2.1021 (10)	C15—H15B	0.9900
Co1—O31	2.1157 (9)	C16—H16A	0.9900
Co1—O21	2.1314 (9)	C16—H16B	0.9900
Co1—N11	2.2489 (11)	O21—C21	1.4446 (15)
N1—C1	1.1610 (18)	O21—H21	0.8400
C1—S1	1.6335 (13)	C21—C22	1.505 (2)
N2—C2	1.1625 (18)	C21—H21A	0.9900
C2—S2	1.6437 (13)	C21—H21B	0.9900
N11—C16	1.4889 (16)	C22—H22A	0.9800
N11—C11	1.4955 (15)	C22—H22B	0.9800
N11—C14	1.4957 (15)	C22—H22C	0.9800
N12—C11	1.4771 (15)	O31—C31	1.4409 (16)
N12—C12	1.4810 (16)	O31—H31	0.8400
N12—C15	1.4878 (16)	C31—C32	1.5157 (19)
N13—C16	1.4705 (16)	C31—H31A	0.9900
N13—C12	1.4783 (16)	C31—H31B	0.9900
N13—C13	1.4855 (16)	C32—H32A	0.9800
N14—C14	1.4640 (16)	C32—H32B	0.9800
N14—C13	1.4648 (17)	C32—H32C	0.9800
N14—C15	1.4695 (16)	O41—C41	1.4339 (16)
C11—H11A	0.9900	O41—H41	0.8400
C11—H11B	0.9900	C41—C42	1.504 (2)
C12—H12A	0.9900	C41—H41A	0.9900
C12—H12B	0.9900	C41—H41B	0.9900
C13—H13A	0.9900	C42—H42A	0.9800
C13—H13B	0.9900	C42—H42B	0.9800
C14—H14A	0.9900	C42—H42C	0.9800
			
N2—Co1—N1	178.73 (4)	N14—C15—N12	111.62 (10)
N2—Co1—O41	90.12 (4)	N14—C15—H15A	109.3
N1—Co1—O41	88.61 (4)	N12—C15—H15A	109.3
N2—Co1—O31	93.75 (4)	N14—C15—H15B	109.3
N1—Co1—O31	86.35 (4)	N12—C15—H15B	109.3
O41—Co1—O31	88.09 (4)	H15A—C15—H15B	108.0
N2—Co1—O21	92.51 (4)	N13—C16—N11	113.06 (10)
N1—Co1—O21	87.27 (4)	N13—C16—H16A	109.0
O41—Co1—O21	86.42 (4)	N11—C16—H16A	109.0
O31—Co1—O21	171.68 (4)	N13—C16—H16B	109.0
N2—Co1—N11	91.69 (4)	N11—C16—H16B	109.0
N1—Co1—N11	89.57 (4)	H16A—C16—H16B	107.8
O41—Co1—N11	177.25 (4)	C21—O21—Co1	123.67 (8)
O31—Co1—N11	93.85 (4)	C21—O21—H21	109.5
O21—Co1—N11	91.43 (4)	Co1—O21—H21	117.9
C1—N1—Co1	176.60 (11)	O21—C21—C22	110.89 (12)
N1—C1—S1	179.67 (14)	O21—C21—H21A	109.5
C2—N2—Co1	168.63 (11)	C22—C21—H21A	109.5
N2—C2—S2	179.00 (12)	O21—C21—H21B	109.5
C16—N11—C11	107.39 (9)	C22—C21—H21B	109.5
C16—N11—C14	107.64 (10)	H21A—C21—H21B	108.0
C11—N11—C14	107.18 (10)	C21—C22—H22A	109.5
C16—N11—Co1	108.63 (7)	C21—C22—H22B	109.5
C11—N11—Co1	115.68 (7)	H22A—C22—H22B	109.5
C14—N11—Co1	110.02 (7)	C21—C22—H22C	109.5
C11—N12—C12	108.83 (10)	H22A—C22—H22C	109.5
C11—N12—C15	108.14 (10)	H22B—C22—H22C	109.5
C12—N12—C15	108.38 (10)	C31—O31—Co1	129.48 (8)
C16—N13—C12	107.78 (10)	C31—O31—H31	109.5
C16—N13—C13	108.23 (10)	Co1—O31—H31	111.1
C12—N13—C13	108.07 (10)	O31—C31—C32	111.56 (11)
C14—N14—C13	109.17 (10)	O31—C31—H31A	109.3
C14—N14—C15	108.43 (10)	C32—C31—H31A	109.3
C13—N14—C15	108.26 (10)	O31—C31—H31B	109.3
N12—C11—N11	111.76 (10)	C32—C31—H31B	109.3
N12—C11—H11A	109.3	H31A—C31—H31B	108.0
N11—C11—H11A	109.3	C31—C32—H32A	109.5
N12—C11—H11B	109.3	C31—C32—H32B	109.5
N11—C11—H11B	109.3	H32A—C32—H32B	109.5
H11A—C11—H11B	107.9	C31—C32—H32C	109.5
N13—C12—N12	111.64 (10)	H32A—C32—H32C	109.5
N13—C12—H12A	109.3	H32B—C32—H32C	109.5
N12—C12—H12A	109.3	C41—O41—Co1	128.97 (8)
N13—C12—H12B	109.3	C41—O41—H41	109.5
N12—C12—H12B	109.3	Co1—O41—H41	121.3
H12A—C12—H12B	108.0	O41—C41—C42	112.33 (13)
N14—C13—N13	112.16 (10)	O41—C41—H41A	109.1
N14—C13—H13A	109.2	C42—C41—H41A	109.1
N13—C13—H13A	109.2	O41—C41—H41B	109.1
N14—C13—H13B	109.2	C42—C41—H41B	109.1
N13—C13—H13B	109.2	H41A—C41—H41B	107.9
H13A—C13—H13B	107.9	C41—C42—H42A	109.5
N14—C14—N11	112.31 (10)	C41—C42—H42B	109.5
N14—C14—H14A	109.1	H42A—C42—H42B	109.5
N11—C14—H14A	109.1	C41—C42—H42C	109.5
N14—C14—H14B	109.1	H42A—C42—H42C	109.5
N11—C14—H14B	109.1	H42B—C42—H42C	109.5
H14A—C14—H14B	107.9		

### \ Tris(ethanol-κO)(hexamethylenetetramine-κN)bis(thiocyanato-\ κN)cobalt(II) (2) Hydrogen-bond geometry (Å, º)

**Table:**

*D*—H···*A*	*D*—H	H···*A*	*D*···*A*	*D*—H···*A*
C12—H12*A*···S2^i^	0.99	2.87	3.6586 (13)	137
C12—H12*B*···S1^ii^	0.99	2.92	3.8813 (13)	164
C15—H15*A*···S1^iii^	0.99	2.99	3.9387 (13)	161
C15—H15*B*···S2^iv^	0.99	2.94	3.7110 (13)	135
C16—H16*A*···O21	0.99	2.54	3.1009 (16)	116
C16—H16*B*···N1	0.99	2.47	3.1083 (17)	122
O21—H21···N13^ii^	0.84	2.03	2.8424 (14)	161
C22—H22*C*···S1^v^	0.98	3.02	3.9559 (16)	161
O31—H31···N12^vi^	0.84	1.96	2.7969 (14)	172
O41—H41···S2^vi^	0.84	2.37	3.2080 (10)	174

Symmetry codes: (i) −*x*+1/2, *y*+1/2, −*z*+1/2; (ii) −*x*+1, −*y*+1, −*z*+1; (iii) *x*−1/2, −*y*+1/2, *z*−1/2; (iv) *x*+1/2, −*y*+1/2, *z*−1/2; (v) *x*−1, *y*, *z*; (vi) *x*+1/2, −*y*+1/2, *z*+1/2.

## References

[bb1] Bai, Y., Shang, W.-L., Dang, D.-B., Sun, J.-D. & Gao, H. (2009). *Spectrochim. Acta Part A*, **72**, 407–411. 10.1016/j.saa.2008.10.033 19062330

[bb2] Bai, Y., Shang, W.-L., Zhang, F.-L., Pan, X.-J. & Niu, X.-F. (2007). *Acta Cryst.* E**63**, m2628.

[bb3] Böhme, M., Jochim, A., Rams, M., Lohmiller, T., Suckert, S., Schnegg, A., Plass, W. & Näther, C. (2020). *Inorg. Chem.* **59**, 5325–5338. 10.1021/acs.inorgchem.9b03357 32091883

[bb4] Brandenburg, K. & Putz, H. (1999). *DIAMOND*. Crystal Impact GbR, Bonn, Germany.

[bb5] Ceglarska, M., Böhme, M., Neumann, T., Plass, W., Näther, C. & Rams, M. (2021). *Phys. Chem. Chem. Phys.* **23**, 10281–10289. 10.1039/d1cp00719j 33903874

[bb6] Chakraborty, J., Samanta, B., Rosair, G., Gramlich, V., Salah El Fallah, M., Ribas, J., Matsushita, T. & Mitra, S. (2006). *Polyhedron*, **25**, 3006–3016.

[bb7] Czubacka, E., Kruszynski, R. & Sieranski, T. (2012). *Struct. Chem.* **23**, 451–459.

[bb8] Dolomanov, O. V., Bourhis, L. J., Gildea, R. J., Howard, J. A. K. & Puschmann, H. (2009). *J. Appl. Cryst.* **42**, 339–341.

[bb10] Groom, C. R., Bruno, I. J., Lightfoot, M. P. & Ward, S. C. (2016). *Acta Cryst.* B**72**, 171–179. 10.1107/S2052520616003954 PMC4822653 27048719

[bb11] Jin, Y., Che, Y. X. & Zheng, J. M. (2007). *J. Coord. Chem.* **60**, 2067–2074.

[bb12] Jochim, A., Rams, M., Böhme, M., Ceglarska, M., Plass, W. & Näther, C. (2020). *Dalton Trans.* **49**, 15310–15322. 10.1039/d0dt03227a 33118568

[bb13] Krebs, C., Jess, I., Ceglarska, M. & Näther, C. (2022). *Acta Cryst.* E**78**, 66–70. 10.1107/S2056989021013281 PMC8739191 35079427

[bb14] Krebs, C., Jess, I. & Näther, C. (2021). *Acta Cryst.* E**77**, 1120–1125. 10.1107/S2056989021010422 PMC8587972 34868648

[bb15] Li, J., Meng, S., Zhang, J., Song, Y., Huang, Z., Zhao, H., Wei, H., Huang, W., Cifuentes, M. P., Humphrey, M. G. & Zhang, C. (2012). *CrystEngComm*, **14**, 2787–2796.

[bb16] Li, X.-L., Niu, D.-Z. & Lu, Z.-S. (2007). *Acta Cryst.* E**63**, m2478.

[bb17] Liu, Q., Xi, H.-T., Sun, X.-Q., Zhu, J.-F. & Yu, K.-B. (2002). *Chin. J. Struct. Chem.* **21**, 355–359.

[bb18] Lu, J., Liu, H.-T., Zhang, X.-X., Wang, D.-Q. & Niu, M.-J. (2010). *Z. Anorg. Allg. Chem.* **636**, 641–647.

[bb19] Mautner, F. A., Traber, M., Fischer, R. C., Torvisco, A., Reichmann, K., Speed, S., Vicente, R. & Massoud, S. S. (2018). *Polyhedron*, **154**, 436–442.

[bb20] Näther, C., Wöhlert, S., Boeckmann, J., Wriedt, M. & Jess, I. (2013). *Z. Anorg. Allg. Chem.* **639**, 2696–2714.

[bb21] Prananto, Y. P., Urbatsch, A., Moubaraki, B., Murray, K. S., Turner, D. R., Deacon, G. B. & Batten, S. R. (2017). *Aust. J. Chem.* **70**, 516–528.

[bb22] Rams, M., Jochim, A., Böhme, M., Lohmiller, T., Ceglarska, M., Rams, M. M., Schnegg, A., Plass, W. & Näther, C. (2020). *Chem. Eur. J.* **26**, 2837–2851. 10.1002/chem.201903924 PMC7078958 31702081

[bb23] Rigaku OD (2021). *CrysAlis PRO*. Rigaku Oxford Diffraction.

[bb24] Shang, W.-L., Bai, Y., Ma, C.-Z. & Li, Z.-M. (2008). *Acta Cryst.* E**64**, m1184–m1185. 10.1107/S160053680802357X PMC2960658 21201625

[bb25] Sheldrick, G. M. (2015*a*). *Acta Cryst.* A**71**, 3–8.

[bb28] Sheldrick, G. M. (2015*b*). *Acta Cryst.* C**71**, 3–8.

[bb29] Shi, J.-M., Chen, J.-N. & Liu, L.-D. (2006). *Pol. J. Chem.* **80**, 1909–1913.

[bb30] Suckert, S., Rams, M., Böhme, M., Germann, L. S., Dinnebier, R. E., Plass, W., Werner, J. & Näther, C. (2016). *Dalton Trans.* **45**, 18190–18201. 10.1039/c6dt03752f 27796392

[bb31] Wellm, C., Majcher-Fitas, A., Rams, M. & Näther, C. (2020). *Dalton Trans.* **49**, 16707–16714. 10.1039/d0dt03428b 33169760

[bb32] Werner, J., Rams, M., Tomkowicz, Z. & Näther, C. (2014). *Dalton Trans.* **43**, 17333–17342. 10.1039/c4dt02271h 25318637

[bb33] Werner, J., Tomkowicz, Z., Rams, M., Ebbinghaus, S. G., Neumann, T. & Näther, C. (2015). *Dalton Trans.* **44**, 14149–14158. 10.1039/c5dt01898f 26182402

[bb34] Westrip, S. P. (2010). *J. Appl. Cryst.* **43**, 920–925.

[bb35] Zhang, Y., Li, J., Xu, H., Hou, H., Nishiura, M. & Imamoto, T. (1999). *J. Mol. Struct.* **510**, 191–196.

